# SORBS1 suppresses tumor metastasis and improves the sensitivity of cancer to chemotherapy drug

**DOI:** 10.18632/oncotarget.12851

**Published:** 2016-10-24

**Authors:** Lele Song, Renxu Chang, Cheng Dai, Yanjun Wu, Jingyu Guo, Meiyan Qi, Wu Zhou, Lixing Zhan

**Affiliations:** ^1^ Key Laboratory of Food Safety Research, Institute for Nutritional Sciences, Shanghai Institutes for Biological Sciences, Chinese Academy of Sciences, Shanghai 200031, China; ^2^ University of the Chinese Academy of Sciences, Shanghai 200031, China; ^3^ Department of Medicine, College of Medicine and Health, Lishui University, Lishui 323000, China

**Keywords:** SORBS1, migration, invasion, JNK, cisplatin sensitivity

## Abstract

Tumor metastasis and invasion are both hallmarks of cancer malignancy and the leading cause of cancer death. Here we show that the adaptor protein SORBS1 (Sorbin and SH3 domain-containing protein 1, also known as CAP/ponsin) is expressed at low levels in clinical cancer samples. In addition, low-level expression of SORBS1 was significantly associated with poor clinical outcomes and the increased tumor cell invasive capacity in breast cancer patients. We demonstrate that depletion of SORBS1 increases protrusions and filopodium-like protrusions (FLPs) formation, as well as the migratory and invasive abilities of cancer cells, via activation of JNK/cJun. Furthermore, silencing of *SORBS1* promotes the epithelial-to-mesenchymal transition (EMT) process and attenuates chemical drug sensitivity especially that to cisplatin, by inhibition of p53 in breast cancer cells. Thus, we illustrate that SORBS1 is a potential inhibitor of metastasis in cancer and may be a promising target in chemotherapy.

## INTRODUCTION

Metastasis is responsible for as much as 90% of cancer-associated mortality; this process remains the most poorly understood component of cancer pathogenesis. Previous studies have shown that the typical characteristics of malignancy involve enhanced migratory and invasive abilities and morphological changes of cells via reorganization of cytoskeleton and the disordering of cell-cell/extracellular matrix (ECM) adhesions [1–4]. Additionally, epithelial-to-mesenchymal transition (EMT) is a potential mechanism by which tumor cells gain metastatic features [2, 4–6]. Recently, several reports revealed that filopodium-like protrusions (FLPs) can facilitate the proliferation of cancer cells that infiltrate into the parenchyma of foreign tissues following egress from the primary site [4, 7]. Furthermore, research on the underlying molecular mechanisms demonstrate that both adhesion molecules (for example, E-cadherin [8, 9], N-cadherin [9], vimentin [10] and integrins [11]) and adaptor proteins (for example, vinculin [12], afadin [13], paxillin [14], Grb2 [15], and ArgBP2 [16]) have effects on invasion and metastasis via changes in actin cytoskeleton, cell-cell/ECM adhesions, or EMT. Although the basic knowledge related to this process has been explored for long time, the underlying key elements are largely unknown and the functions of many suspected regulators remain to be verified.

SORBS1 (Sorbin and SH3 domain-containing protein 1, also known as CAP/ponsin) is an adaptor protein that belongs to SOHO family [17, 18]. It has been reported that SORBS1 localizes at both cell-ECM and cell-cell adhesion sites. Additional studies reveal that SORBS1 can bind to signaling molecules (e.g., c-Cbl [19], c-Abl [20, 21], and insulin reporter (IR) [20]) to regulate glucose transport [22, 23], transcription activity [18], and insulin signaling [20, 21]. Moreover, SORBS1 can interact with cytoskeleton regulators (e.g., vinculin [24], paxillin [25], nectin, afadin [26]) to modulate cell adhesion [27], cytoskeleton organization [18, 28], cell spreading, and cell motility [27]. In addition, SORBS1 attenuates cell motility via inhibition of PAK/MEK/ERK activity in REF52 fibroblasts [27]. Furthermore, SORBS1 has been described as a new target of p53 in a colon cancer cell line model [29].

In the present study, we explored whether SORBS1 plays a critical role in tumor migration, invasion, and tumorigenesis. We employed data obtained from the Oncomine and Kaplan Meier-plotter websites to investigate the effects of SORBS1 loss on cancer metastasis and chemical drug sensitivity. Our data proved that the low-level SORBS1 has clinically positive correlation with the progression of breast and lung cancers. Additionally, we demonstrated, using three-dimensional (3D) cell culture and *in vivo* models, that the depletion of SORBS1 enhances the migratory and invasive abilities and increases FLPs formation via activation of JNK/c-Jun signaling in cancer cells. We then demonstrated that SORBS1 is positively correlated with the drug sensitivity of breast cancer cells via increased accumulation of p53 protein after chemical drug treatment. In conclusion, our work illustrates that SORBS1 impedes cancer-metastasis and sensitizes cancer cells to chemotherapy. We expect that SORBS1 may be a useful marker and/or target for designing new therapeutic strategies and for evaluating the prognostic outcome in patients with breast cancer or lung cancer.

## RESULTS

### SORBS1 is present at a lower level in human breast cancer

**Figure 1:**
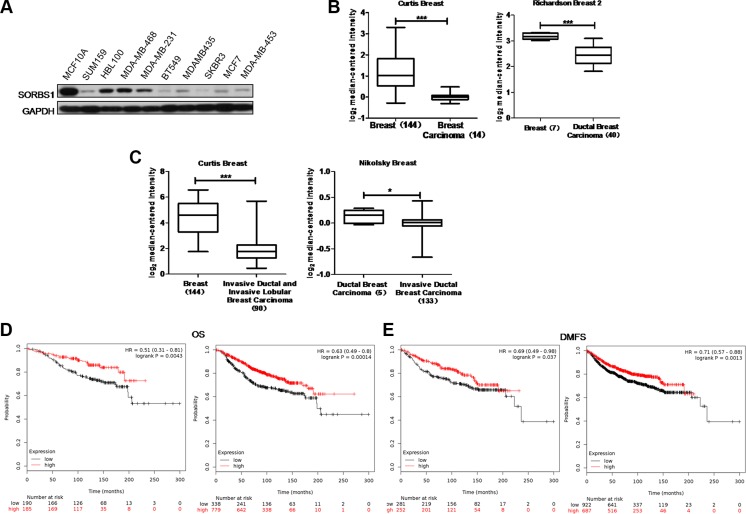
SORBS1 is present at a lower level in human breast cancer (**A**) Western blots was performed to detect SORBS1 levels in normal human mammary gland epithelial cell line MCF 10A and nine other human breast cancer cell lines. (**B**) Box plots comparing levels of *SORBS1* mRNA in normal human breast tissues and breast carcinomas (left panel) or ductal breast carcinoma tissues (right panel) in published data sets from Oncomine. ***P < 0.001, Student's *t-*test. (**C**) mRNA levels of *SORBS1* in normal breast tissues vs invasive ductal / lobular breast carcinomas tissues in the case of Curtis Breast, or mRNA levels of *SORBS1* in ductal breast carcinomas vs invasive ductal breast carcinoma in Nikolsky Breast in published data sets from Oncomine. *P < 0.05, Student's *t-*test. (**D** and **E**) Kaplan-Meier survival analysis for assessment of overall survival (OS) (D) and distant metastasis-free survival (DMFS) (E) in breast cancer patients with (right panel) or without (left panel) systemic treatment which classified by relative (high or low) tumor *SORBS1* level. Survival curves were generated by using the Kaplan-Meier Plotter online tool based on data stratified based on the best cut-off. Curves were compared by hazard ratios (HR) and *p* values (log rank *p*).

To explore the function of SORBS1 in breast tumorigenesis, we investigated the protein levels of SORBS1 in breast cancer cells. We found that levels of SORBS1 were lower in the majority of breast cancer cells compared to the level in the normal mammary epithelial cell line MCF10A (Figure 1A). Consistent with those results, analyses of two independent Oncomine data-sets, *Richardson Breast 2* and *Curtis Breast*, revealed that *SORBS1* mRNA levels were lower in breast carcinoma patient samples (*Richardson Breast 2*,*N* = 40; *Curtis Breast*, *N* = 14) compared with those in normal breast samples (*Richardson Breast 2*, *N* = 7; *Curtis Breast*, *N* = 144) (Figure 1B, Supplementary Table S1). Furthermore, analyses of the *Curtis Breast* and *Nikolsky Breast* data-sets in Oncomine also suggested that the lower levels of *SORBS1* were significantly correlated with the higher invasive ability in ductal and lobular breast carcinoma (Figure 1C, Supplementary Table S2). To further investigate whether SORBS1 correlated with prognosis of patients with breast cancer, an online Kaplan Meier-plotter website [30] was used for analyses. Among patients with or without systemic treatment, the probability of overall survival (OS) and distant metastasis-free survival (DMFS) was dramatically worse in patients with lower SORBS1 expression levels than that in patients with higher SORBS1 expression levels (Figure 1D–1E). All of these analyses indicated that decreased levels of SORBS1 have significantly positive correlation with poor clinical outcomes and more malignant phenotype in breast cancer patients.

We extended these observations in breast cancer by examining the levels of SORBS1 in lung cancer cell lines, analyzing the Oncomine gene expression data-sets for lung carcinoma and OS of lung cancer patients (based on analyses obtained from the Kaplan Meier-plotter website [31]). Consistent with the results in breast cancer, lower *SORBS1* mRNA levels also were detected in lung cancer cell lines and lung cancer samples (Supplementary Figure S1A–S1B, Supplementary Table S3). In addition, patients harboring tumors with lower SORBS1 expression levels (*N* = 966) showed decreased OS probabilities compared to those in patients harboring tumors with higher SORBS1 expression levels (*N* = 960) (Supplementary Figure S1C).

### Loss of SORBS1 increases breast cancer cells migration and invasion properties both *in vivo* and *in vitro*

**Figure 2:**
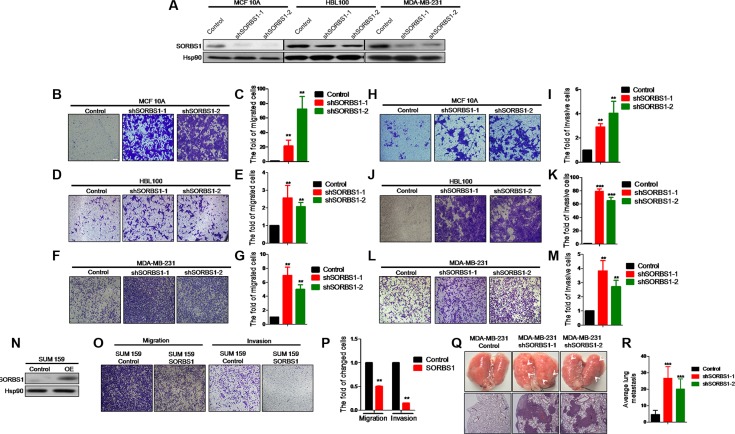
Loss of SORBS1 increases breast cancer cells migration and invasion properties both *in vivo* and *in vitro* (**A**) Western blot results to show the knockdown efficiency of *SORBS1* gene in MCF10A, HBL100 and MDA-MB-231 cell lines. (**B**, **D** and **F**) Migration transwell assay was done in MCF 10A Control (B), HBL100 Control (D), MDA-MB-231 Control (F) and corresponding shSORBS1 (respectively named as shSORBS1-1 and shSORBS1-2) cell lines, in which cells (B and D: 4 × 10^4^ cells/well, F: 3 × 10^4^ cells/well) were cultured for 24 hours. (**C**, **E** and **G**) Quantitative results are respectively illustrated for panel B, D and F. (**H**, **J** and **L**) Invasion transwell assay was done in MCF 10A control (H), HBL100 Control (J), MDA-MB-231 Control (L) and corresponding shSORBS1 cell lines, in which cells (H and J: 8 × 10^4^ cells/well, L: 6 × 10^4^ cells/well) were cultured for 24 hours. (**I**, **K** and **M**) Quantitative results are respectively illustrated for panel H, J and L. (**N**) SUM 159 cells were efficiently transfected with a construct encoding SORBS1 or with a control plasmid. (**O** and **P**) Transwell assay was performed in SUM159 Control and SUM 159 SORBS1 (OE), in which cells (migration, 3 × 10^4^ cells/well; invasion, 6 × 10^4^ cells/well) were cultured for 24 hours. (P) Quantitative results from panel O. All of images were taken by 10× objective lens. The number of cells was counted from at least four independent microscopic fields. (**Q)** Lung tissues were photographed, fixed and stained with haematoxylin and eosin (HE). White arrows indicated the lung metastatic lesions. (**R**) The number of metastatic lesions in each specimen was counted by randomly selected four fields under light microscope. Data are shown as mean ± s.d. (*n* = 4), **P < 0.01, ***P < 0.001, Student's *t-*test.

To define the effects of *SORBS1* deficiency on breast cancer progression, we used virus-mediated RNA interference to “knock down” the expression of *SORBS1* in MCF10A, HBL100, and MDA-MB-231 cell lines. Western blot analysis confirmed that SORBS1 were decreased in each of these cell lines (Figure 2A). Subsequent analysis indicated that loss of SORBS1 had no significant impact on cell proliferation (Supplementary Figure S2A–S2C). The result from an *in vitro* wound-healing assay using MCF10A showed that MCF10A shSORBS1 cell lines (designated MCF10A shSORBS1-1 and MCF10A shSORBS1-2) displayed higher motility than the parent control cell line, MCF10A (Supplementary Figure S2D). Transwell assays in MCF10A, MDA-MB-231, and HBL100 cell lines with knockdown of SORBS1 demonstrated that loss of SORBS1 resulted in increased migration (Figure 2B–2G) and invasion (Figure 2H–2M). To verify the suppressive role of SORBS1 in tumor migration and invasion, we also tested the effect of overexpressing *SORBS1* in another human breast cancer cell line, SUM159. Notably, elevation of SORBS1 expression inhibited the migratory and invasive abilities of SUM159 cells (Figure 2N-2P). In extension of our work with breast cancer cell lines, we also found that knockdown of SORBS1 in a lung cancer cell line (CRL-1848^TM^) also enhanced migratory and invasive abilities (Supplementary Figure S3).

Next, we explored the impact of SORBS1 on breast cancer malignancy and metastasis *in vivo* using a mouse model in which MDA-MB-231 Control and MDA-MB-231 shSORBS1 (shSORBS1-1 and shSORBS1-2) cell lines were implanted by intravenous via the tail. Consistent with the *in vitro* results, MDA-MB-231 shSORBS1-injected animals exhibited (at Day 21 post-injection) a significantly elevated number of micro-metastases in the lung compared to animals injected with the MDA-MB-231 Control cell line (Figure 2Q–2R). Collectively, our data demonstrated SORBS1 impedes migration and invasion *in vitro* and suppresses metastasis *in vivo*.

### Loss of SORBS1 expression triggers increased protrusion formation and abundant FLPs in 3D culture

**Figure 3:**
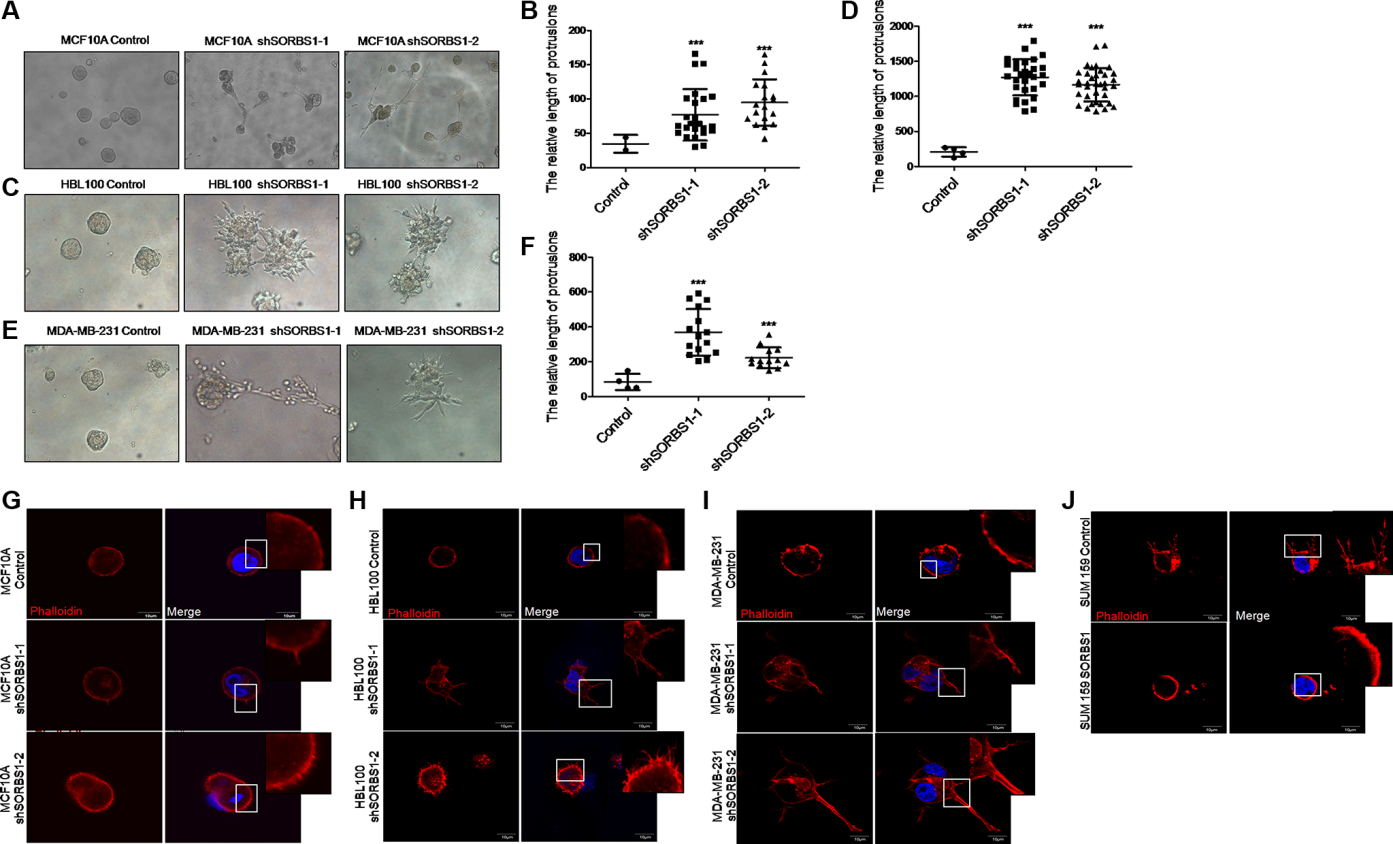
Loss of SORBS1 expression triggers increased protrusion formation and abundant FLPs in 3D culture (**A**, **C** and **E**) Phase-contrast images of MCF 10A cells (A), HBL100 cells (C) or MDA-MB-231 cells (E) stably expressing control or shSORBS1 and grown as 3D culture (Day 10). (**B**, **D** and **F**) Quantitative results of the length of protrusions were illustrated respectively for panel A, C and E. Results are presented as mean ± s.d. (*n* = 3), ***P < 0.001, Student's *t-*test. (**G**–**I**) Filopodium like protrusions (FLPs) formation of MCF 10A (G), HBL100 (H), MDA-MB-231 (I) control cells and corresponding two shSORBS1 cell lines. (**J**) FLPs formation of SUM159 Control cells and forced SORBS1 expression (OE) cells. In FLPs formation assay, 2 × 10^3^ cells were cultured in 3D matrigel and the location of DAPI (blue) and phalloidin (red) were determined at 24 hours. The white arrows indicated FLPs. The experiment was repeated for three times. Scale bars, 20 μm.

3D culture recreates the architecture of epithelial tissue growth *in vitro*, and so is a powerful tool for investigating the molecular mechanisms of tumor growth. Therefore, we used 3D culture to extend our analyses from 2D (plate) growth. When assessed in a 3D culture system, *SORBS1*-silenced MCF10A, HBL100 and MDA-MB-231 cells exhibited more malignant phenotypes compared with controls including increases in both the numbers of protrusions and the lengths of those protrusions (Figure 3A–3F). Furthermore, we observed the formation of filopodium-like protrusions (FLPs) and the data showed that the cells lack of SORBS1 developed more abundant FLPs than the control cells (Figure 3G–3I). In contrast, forced expression of *SORBS1* in SUM159 led to decreased numbers of FLPs (Figure 3J). Thus, under 3D culture conditions, the level of SORBS1 expression is inversely correlated with the development of a more malignant phenotype in breast cancer cell lines.

### Knockdown of SORBS1 induces epithelial-to-mesenchymal transition in breast cancer cells

**Figure 4:**
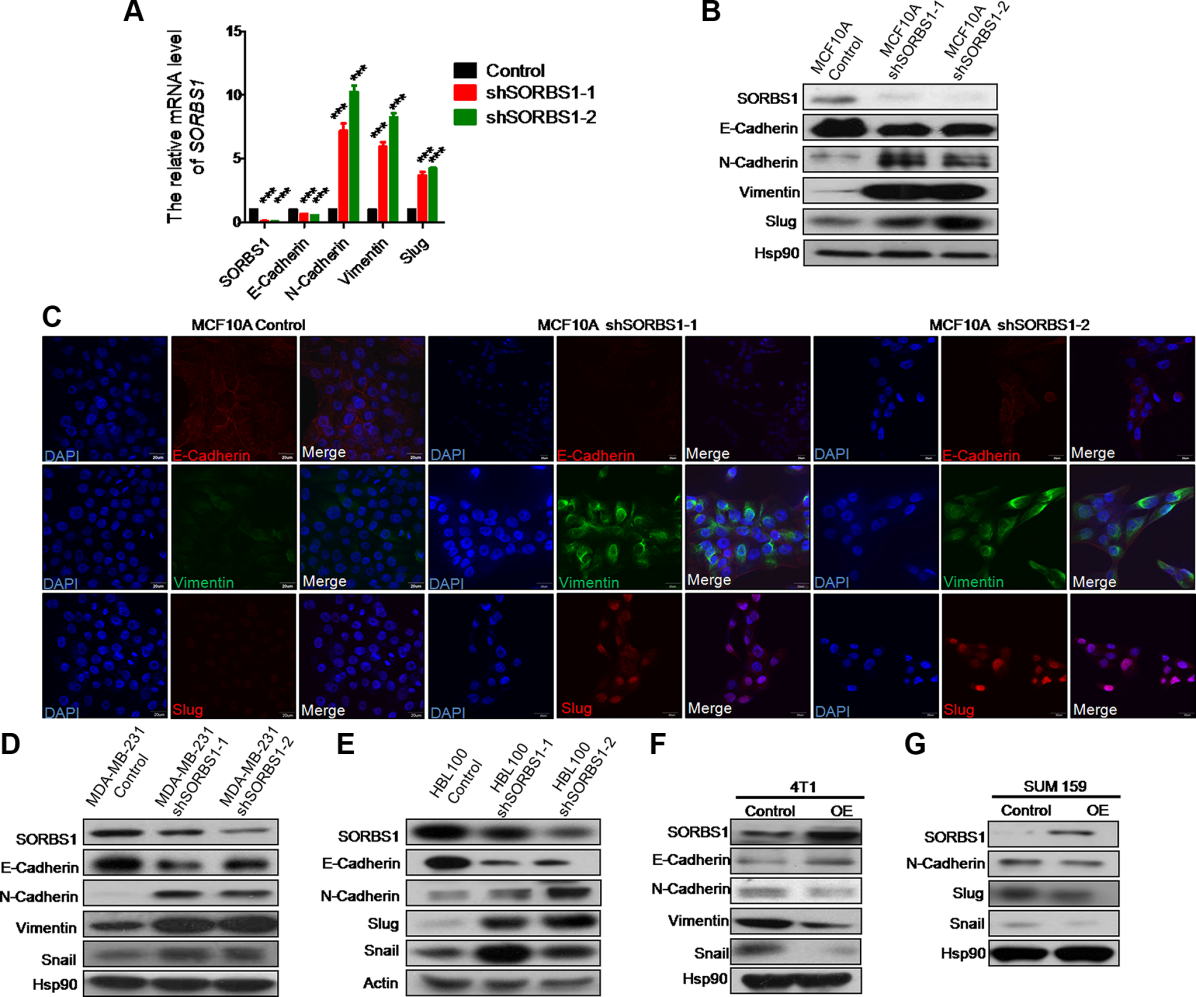
Knockdown of SORBS1 induces epithelial-to-mesenchymal transition in breast cancer cells (**A**) By using of quantitative real time PCR (qPCR),mRNA of *SORBS1* and EMT markers (*E-cadherin*, *N-cadherin*, *vimentin*, *Slug*) were detected in MCF 10A control and two shSORBS1 cell lines. Data are shown as mean ± s.d. (*n* = 3), ***P < 0.001, Student's *t-*test. (**B**) The protein levels of SORBS1 and EMT markers (E-cadherin, N-cadherin, vimentin, and Slug) were analyzed by western blot in MCF 10A Control and shSORBS1 cell lines. (**C**) Immunofluorescence staining images show the protein patterns of EMT markers (E-cadherin, vimentin, Slug) in MCF 10A Control and shSORBS1 cell lines. Scale bar, 20 μm. (**D** and **E**) The protein levels of SORBS1 and EMT markers (E-cadherin, N-cadherin, vimentin, Slug, Snail) were detected by western blot in MDA-MB-231 Control or shSORBS1 (D) and HBL100 Control or shSORBS1 (E) cell lines. (**F** and **G**) Western blot was performed to analyze the expression of SORBS1 and EMT markers (E-cadherin, N-cadherin, vimentin, Snail) in 4T1 (F) and SUM 159 (G) transiently transfected with 1 μg of control vector or *SORBS1* cDNA.

To explore the biologic basis for metastasis in SORBS1-depleted cells, we monitored whether SORBS1 depletion induced the propensity to undergo the process of EMT. Whether assessed by mRNA level or protein level, depletion of SORBS1 led to decreased expression of an epithelial marker (E-cadherin) and increased expression of mesenchymal markers (including N-cadherin, vimentin, and Slug) in MCF 10A shSORBS1 cells compared with expression in control cells (Figure 4A–4B). Immunofluorescent (IF) staining for EMT markers E-cadherin, vimentin, and Slug in MCF10A cell lines with and without SORBS1 knockdown confirmed these effects on EMT (Figure 4C). In further confirmation, depletion of SORBS1 led to dramatic increases in EMT in breast cancer cell lines MDA-MB-231 and HBL100 (Figure 4D–4E). Conversely, we observed attenuation of the EMT phenotype in 4T1 (a highly metastatic mouse breast cancer cell line) and in SUM159 with forced expression of *SORBS1* (Figure 4F–4G). Furthermore, our results indicated that there was a negative correlation between SORBS1 expression level and EMT pattern in MCF7 (Supplementary Figure S4A). Altogether, these results demonstrate that SORBS1 suppresses EMT in breast cancer cells.

We hypothesized that SORBS1 plays the similar role in lung cancer cells. Indeed, we obtained the expected results in non-small-cell lung cancer cells CRL-1848^TM^ (Supplementary Figure S4B–S4C). Considered together, these data conclusively demonstrate that depletion of SORBS1 promotes EMT in cancer cell lines.

### SORBS1 inhibits JNK signaling pathway

**Figure 5:**
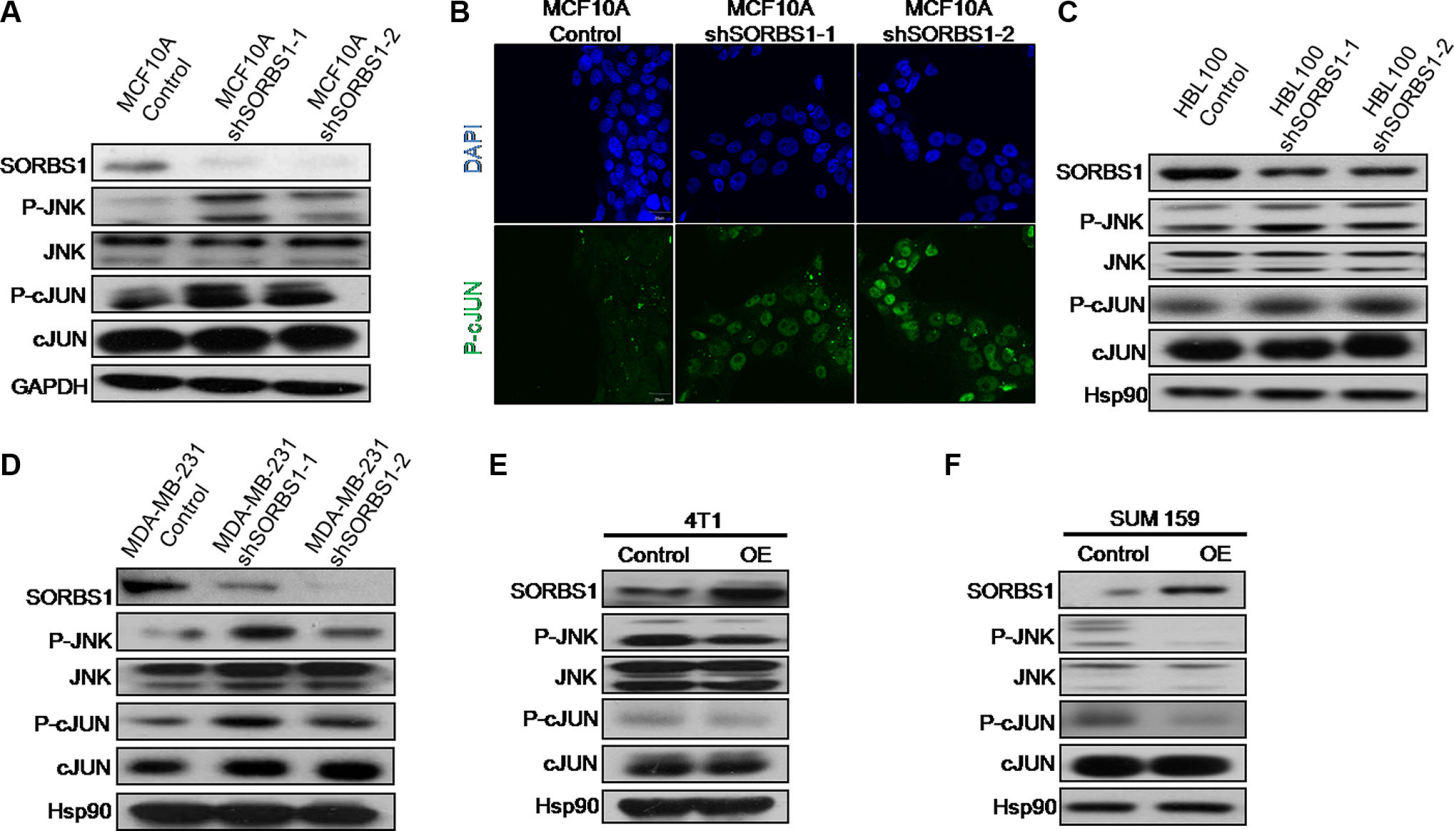
SORBS1 inhibits JNK signaling pathway (**A**) JNK, p-JNK, c-Jun, p-c-Jun and SORBS1 were analyzed by the Western blotting assay in MCF 10A Control and MCF 10A shSORBS1 cell lines. (**B**) P-c-Jun staining were detected by immunofluorescence in MCF10A Control and MCF10A shSORBS1 cells. Scale bar, 20 μm. (**C** and **D**) Western blot was performed to detect the levels of JNK, p-JNK, c-Jun, p-c-Jun and SORBS1 in HBL100 Control cells (C), MDA-MB-231 Control cells (D) and respectively stably expressing the shSORBS1 cell lines. (**E** and **F**) The levels of JNK, p-JNK, c-Jun, p-c-Jun and SORBS1 in Control and SORBS1 (OE) cells were detected by western blot in 4T1 (E) and SUM159 (F).

Previous work has shown that SORBS1 has a negative impact on cell motility via inhibiting the ERK pathway [27]. However, in our own research, we have not detected significant changed in ERK activation in *SORBS1*-silenced cell lines (data not shown). Unexpectedly, we did observe that JNK/c-Jun, a component of the MAPK pathway, was dramatically activated not only in MCF10A shSORBS1 cells (Figure 5A) but also in HBL100 shSORBS1 cells (Figure 5C) and MDA-MB-231 shSORBS1 cells (Figure 5D) when compared with respective control cells. In addition, we observed (by IF staining) increased phosphorylation of c-Jun in MCF10A shSORBS1 cells (Figure 5B). In contrast, overexpression of SORBS1 resulted in decreased activation of JNK and c-Jun in 4T1 and SUM159 (Figure 5E and 5F). Furthermore, we demonstrated that overexpression of SORBS1 yielded decreased phosphorylation of JNK and c-Jun in MCF7 cells, an effect that exhibited apparent dose dependence (Supplementary Figure S5). Taken together, these results demonstrated that SORBS1 inhibits the activation of JNK and c-Jun in breast cancer cells.

### Inhibition of JNK activity decreases migration, invasion, and FLPs formation in SORBS1 knockdown cells

**Figure 6:**
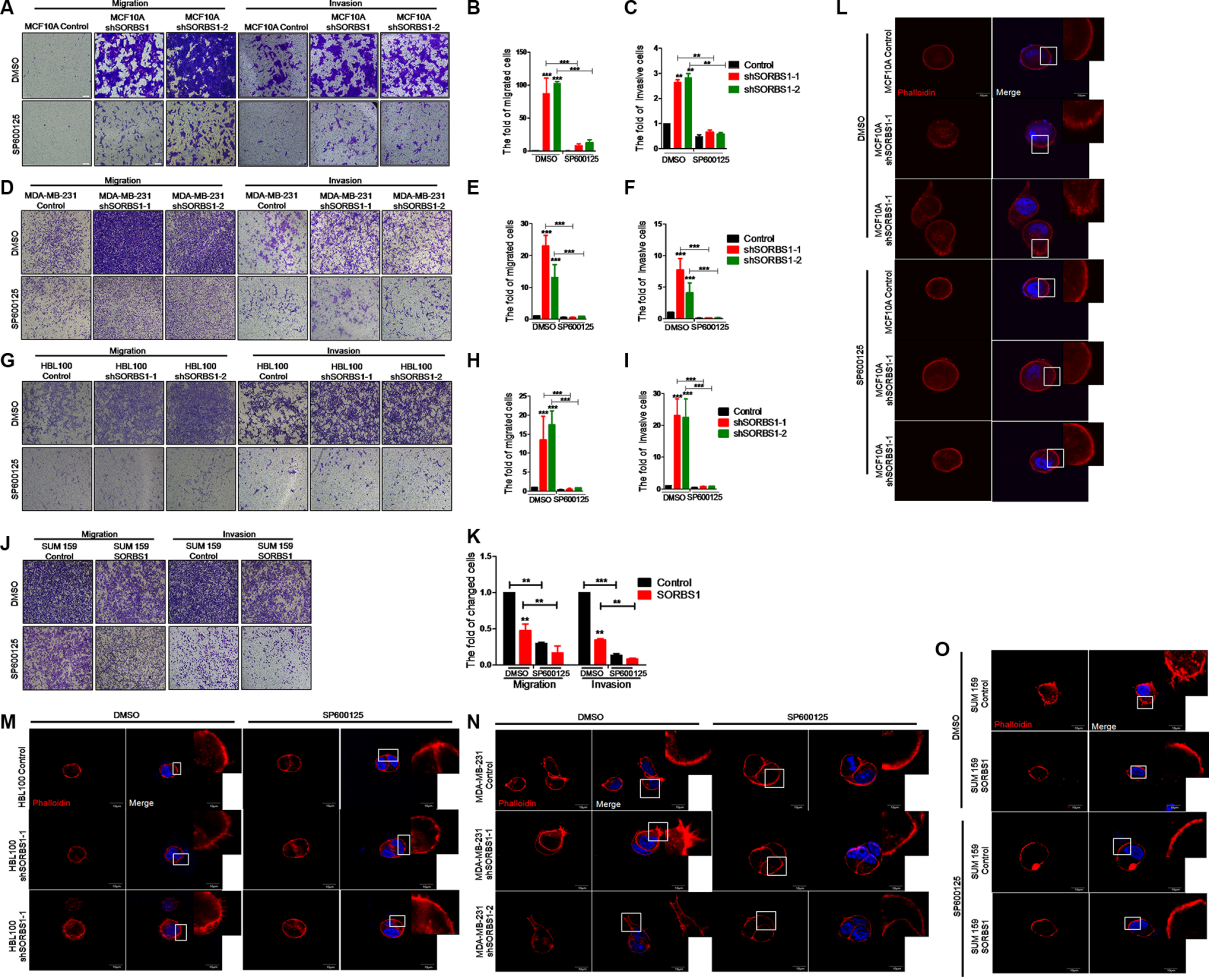
Inhibition of JNK activity decreases migration, invasion and FLPs formation in SORBS1 knockdown cells (**A**, **D** and **G**) Transwell assay were conducted in MCF 10A Control (A), MDA-MB-231 Control (D), HBL100 Control cells (G) and corresponding stably expressing shSORBS1 cells which were treated with DMSO or sp600125 (10 μM) for 24 hours. Migration assay: MCF 10A and HBL100 (4 × 10^4^ cells/well), MDA-MB-231 (3 × 10^4^ cells/well). Invasion assay: MCF 10A and HBL100 (8 × 10^4^ cells/well), MDA-MB-231 (6 × 10^4^ cells). Images were taken with a 10× objective lens. (**B** and **C**) Quantitative results are illustrated for migration and invasion in panel A. (**E** and **F**) Quantitative results from panel D. (**H** and **I**) Quantification of transwell migration and invasion assay from panel G. (**J** and **K**) Transwell assay were conducted in SUM159 Control and SORBS1 overexpressed (OE) cells, in which cells (migration, 3 × 10^4^ cells/well; invasion, 6 × 10^4^ cells/well) were cultured and treated with DMSO or sp600125 (10 μM) for 24 hours. (K) Quantification of transwell migration and invasion assay from panel J. The numbers of cells were counted from at least four independent microscopic fields. Data are shown as mean ± s.d. (*n* = 3), **P < 0.01, ***P < 0.001, Student's *t-*test. (**L**–**O**) FLPs formation of MCF10A shSORBS1 (L), HBL100 shSORBS1 (M), MDA-MB-231 shSORBS1 (N), SUM159 SORBS1 (OE) (O) and corresponding control cell lines. 3 × 10^3^ cells/well were seeded in 3D matrigel and treated with DMSO or sp600125 (10 μM) for 24 hours. The location of DAPI (blue) and phalloidin (red) were determined. The experiment was repeated for three times. Scale bars, 20 μm.

It has been reported that JNK/c-Jun signaling promotes migration and invasion in multiple cancers [32, 33]. Therefore, we next investigated whether SORBS1 inhibits cell migration and invasion via suppression of JNK/c-Jun activation. For this purpose, we employed SP600125, a specific small-molecule inhibitor of JNK kinase activation. Using an *in vitro* wound healing assay, we confirmed that increased cell motility of MCF10A upon knockdown of SORBS1 was attenuated by treatment with SP600125 (Supplementary Figure S6). Notably, the data from transwell assay showed that the enhanced migration and invasion patterns induced by depletion of SORBS1 were rescued in MCF10A shSORBS1 cells treated with SP600125 (Figure 6A–6C). Furthermore, treatment with SP600125 also blocked the migration and invasion in MDA-MB-231 shSORBS1 cell lines (Figure 6D–6F) and HBL100 shSORBS1 cell lines (Figure 6G–6I) compared with the respective untreated shSORBS1 cell lines. These results indicated that SORBS1 suppresses cell migration and invasion via JNK/c-Jun signaling in breast cancer cells. We further investigated that whether SORBS1 regulated FLPs formation by inhibition of JNK activation. Our data showed that SP600125 dramatically abolished the enhanced FLPs formation previously observed upon SORBS1 depletion in MCF 10A and breast cancer cells (Figure 6L–6N). Consistent with those data, SP600125 treatment also suppressed FLPs formation, migration and invasion in the SUM159 (Figure 6J–6KJ, 6O). Considered together, these results illustrated that SORBS1 antagonizes migration, invasion and FLPs formation through JNK/c-Jun pathway.

### SORBS1 promotes cisplatin-related drug sensitivity in breast cancer cells

**Figure 7:**
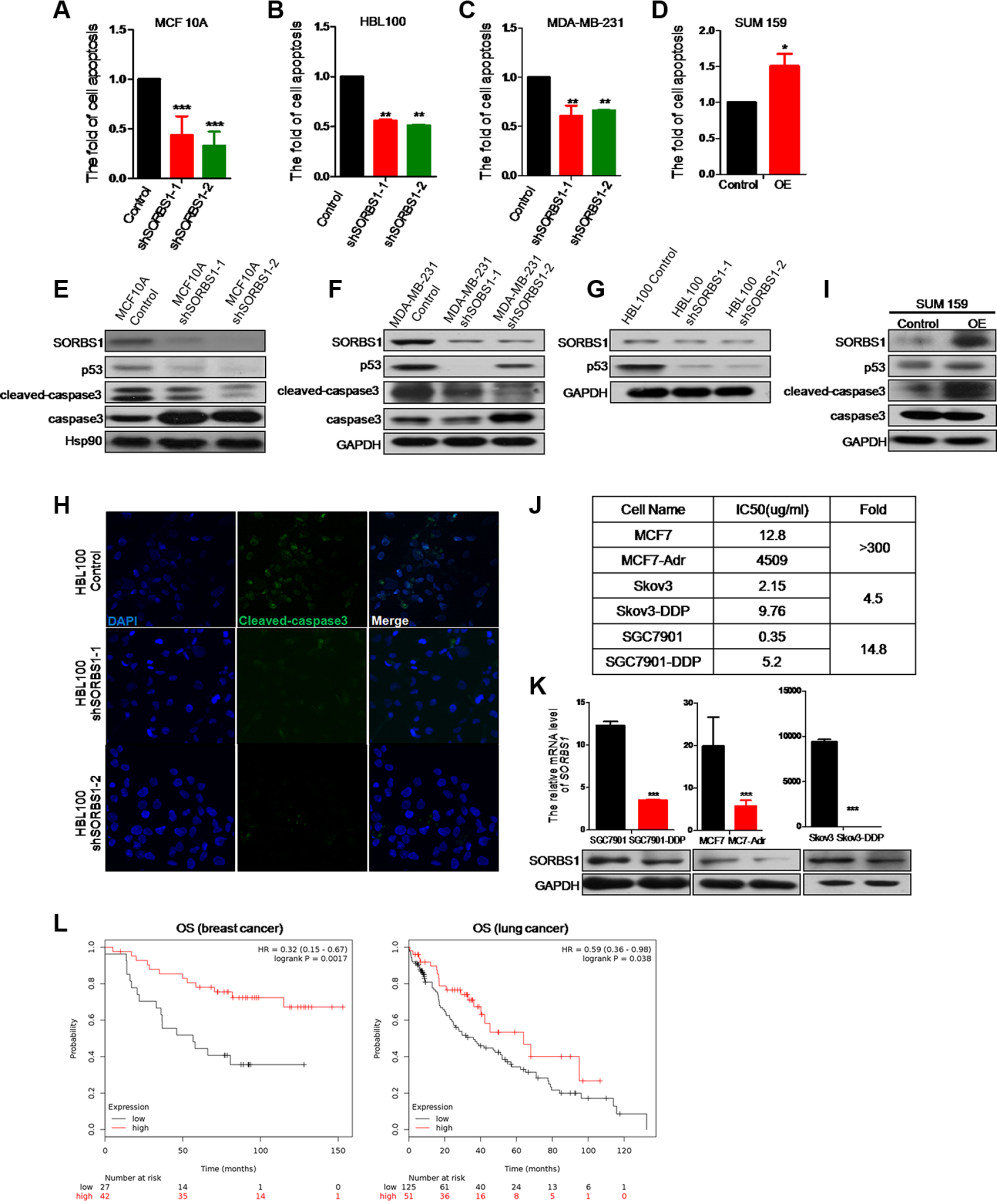
SORBS1 promotes cisplatin-related drug sensitivity in breast cancer cells (**A**–**C**) flow cytometry (FCM) was performed to analyze apoptosis in MCF 10A control (A), HBL100 control (B), MDA-MB-231 control (C) and respectively shSORBS1 cell lines. Cells were treated with 10 ug/ml cisplatin for 24 hours and data are shown as mean ± s.d. (*n* = 3). **P < 0.01, ***P < 0.001, Student's *t-*test. (**D**) SUM159 cells were transiently transfected with 1 μg of vector (Control) or human *SORBS1* (OE). After 24 hours, cells were treated with 10 ug/ml cisplatin for another 24 hours. The rate of apoptosis was analyzed by FCM. Data are shown as mean ± s.d. (*n* = 3). **P < 0.01, Student's *t-*test. (**E**–**G**, **I**) Western blot was conducted to detect the expression of apoptosis related proteins in MCF 10A shSORBS1 (E), MDA-MB-231 shSORBS1 (F), HBL100 shSORBS1 (G), SUM159 SORBS1 (OE) (I) and respectively control cell lines treated with 10 ug/ml cisplatin for 24 hours. (**H**) Immunofluorescence staining was performed to detect the protein patterns of cleaved-caspase3 in HBL100 Control and shSORBS1 cell lines treated with 10 ug/ml cisplatin for 24 hours. Images were taken with a 40× objective lens. (**J**) IC50 values were detected by MTT in MCF7-Adr (anti Adriamycin), Skov3-DDP (anti cisplatin) and SGC7901-DDP (anti cisplatin) and respectively parent cell lines. The IC50values were calculated. The experiment was repeated for three times. (**K**) qPCR and western were performed to analyze the mRNA and protein levels of SORBS1 in MCF7, Skov3, SGC7901 and corresponding chemical drug resistance cell lines. Data are shown as mean ± s.d. (*n* = 3), ***P < 0.001, Student's *t-*test. (**L**) Kaplan-Meier survival analysis for assessment of overall survival (OS) analysis based tumor *SORBS1* level in 69 breast cancer patients with systemic cisplatin chemotherapy (left panel) and 176 chemotherapy treated lung cancer patients (right panel). Survival curves were generated by using the Kaplan-Meier Plotter online tool based on data stratified based on the best cut-off. Curves were compared by hazard ratios (HR) and *p* values (log rank *p*).

Growing evidence suggests that EMT contributes the resistance of cancer cells to cancer drugs [34–37]. We used flow cytometry to measure the potential effects of SORBS1 on the induction of apoptosis by cisplatin, which is clinically one of the most frequently used anti-cancer drugs. Loss of SORBS1 expression significantly decreased cisplatin-induced cellular apoptosis in MCF10A and in breast cancer cell lines MDA-MB-231 and HBL100 (Figure 7A-7C). In contrast, forced expression of SORBS1 in SUM159 and MCF7 remarkably increased the frequency of cellular apoptosis induced by cisplatin (Figure 7D, Supplementary Figure S7). To define the molecular mechanism of these effects, we determined the levels of apoptosis-related molecules (e.g., p53, cleaved-caspase3) in breast cancer cells that had been treated with cisplatin. We observed that the levels of p53 and cleaved-caspase3 activity were dramatically suppressed in cisplatin-treated, SORBS1-depleted cell lines (MCF10 shSORBS1 and MDA-MB-231 shSORBS1) compared to cisplatin-treated control cell lines unaltered in SORBS1 expression (Figure 7E–7F). In confirmation of these data, we further demonstrated that the levels of p53 and cleaved-caspase3 were downregulated in cisplatin-treated HBL100 shSORBS1 cells compared to cisplatin-treated HBL100 control cells (Figure 7G–7H). Conversely, levels of p53 and cleaved-caspase3 were significantly increased in cisplatin-treated SUM159 cells with forced expression SORBS1 compared to cisplatin-treated SUM159 control cells (Figure 7I). Considered together, these data demonstrated that SORBS1 has positive correlation with cisplatin sensitivity via increasing p53 protein levels in breast cancer cells.

To determine whether loss of SORBS1 causes increased resistance to other chemotherapeutic drugs, we assessed the mRNA and protein level of SORBS1 in several commercially obtained cell lines (MCF7-Adr, SGC7901-DDP, and SKBR3-DDP) reported to possess chemical drug resistance. As a first step, we confirmed that each of these strains exhibited elevated (4.5- to 350-fold) IC50s compared to the respective parent lines (Figure 7J). As expected, each of these drug-resistant strains also exhibited lower levels of SORBS1 protein than the respective parent lines (Figure 7K). Furthermore, we used Kaplan Meier-plotter website [31] to define the potential correlation between SORBS1 level and overall survival of patients treated with chemotherapy (based on clinical data from 69 breast cancer patients treated with systemic cisplatin chemotherapy and 176 chemotherapy treated lung cancer patients). The analyses indicated that patients with low-level SORBS1 expression (Breast cancer, *N* = 27; Lung cancer, *N* = 125) exhibited decreased OS probability compared to patients with high-level SORBS1 expression (Breast cancer, *N* = 42; Lung cancer, *N* = 51) (Figure 7L). Therefore, loss of SORBS1 could reduce the sensitivity to cisplatin as well as establish chemotherapeutic drugs resistance in multiple types of cancer cells. Thus, our analyses indicated that SORBS1 deficiency is positively correlated with a poorer response to cancer chemotherapy in multiple cancer types.

## DISCUSSION

This study provides novel insights into the function of SORBS1 in breast and lung cancers. SORBS1 is known to be major negative player in the control of cellular migration of fibroblast cell by down-regulation of PAK/MEK/ERK signaling [27, 28]. However, the role of SORBS1 in tumorigenesis remained largely unknown prior to the work described here. First, we demonstrated that decreased SORBS1 levels are positively correlated with poorer clinical outcomes. Second, we used *in vitro* (2D & 3D culture systems) and *in vivo* (animal model) systems to assess malignancy of cell lines engineered to express SORBS1 at decreased or increased levels. These experiments demonstrated that SORBS1 levels are inversely correlated with migration, invasion, FLPs formation, and EMT activity in both breast and lung cancer cell lines, as well as resistance to chemical drugs such as cisplatin. These experiments also revealed that the inhibition of SORBS1 in cancer cells malignant transformation is mediated by repressed JNK/c-Jun activation, and that the suppression of SORBS1 in drug resistance is mediated by increased p53 level.

To overcome obstacles to invasion, tumor cells may co-opt the EMT process, which may govern morphogenesis in multi-cellular organisms and promote tumorigenesis and metastasis [5, 34]. We observed that depletion of SORBS1 is sufficient for facilitating EMT process. Additionally, prior studies suggest that EMT can encourage the formation of FLPs in 3D culture [7, 38]. Therefore, we hypothesized that SORBS1 might inhibit the formation of FLPs. As we postulated, the formation of FLPs was markedly increased in *SORBS1*-depleted breast cancer cells. We further confirmed the repressive effects of SORBS1 on cancer metastasis *in vivo* using a mouse model of pulmonary metastasis by a breast cancer cell line. These results demonstrated that the *SORBS1* gene not only inhibits EMT activation but also potentially suppresses the process whereby extravasated cancer cells colonize distal sites and develop into metastatic carcinoma.

The MAPK pathway is central mediator of eukaryotic cellular signaling, playing important roles in cancer proliferation, migration, invasion, and metastasis [39–41]. Recent data suggest that SORBS1 may repress cell spreading and migration by inhibiting the activation of RAF/MEK/ERK signaling in fibroblast cells [27]. Therefore, we sought to investigate the underlying molecular mechanism whereby depletion of SORBS1 resulted in the elevated migratory and invasive abilities of cancer cells. In the work reported here, we found that overexpression of SORBS1 inhibited the activation of JNK and the downstream molecule c-Jun, but had no effect on ERK or p38 signaling in breast cancer or lung cancer cells. Previous studies have shown that JNK signaling can stimulate EMT via cooperation with other EMT-related signaling pathways (such as Wnt/TGF-beta) in cancer cells [41–43]. Thus, our data indicated that SORBS1 might impede the EMT process via activation of JNK/c-Jun signaling. Specifically, we demonstrated that *SORBS1* knockdown induced EMT, FLPs formation, migration, and invasion, and that these effects were abrogated by treatment of the same cancer cells with a small-molecule JNK inhibitor.

Drug resistance has been regarded as a major obstacle in cancer treatment, with resistance typically leading to the failure of chemotherapy. Several previous reports have shown that the progression of EMT attenuates cancer cell sensitivity to anti-cancer drugs [34, 35, 37]. As reported here, our studies indicated that SORBS1 inhibits EMT, suggesting that SORBS1 also might impact drug sensitivity. Consistent with that hypothesis, we observed that SORBS1 protein accumulates to significantly lower levels in drug-resistant cancer cell lines compared to the respective parental lines. Moreover, the level of SORBS1 expression was positively correlated with cisplatin sensitivity in breast cancer cells. Additionally, an analysis performed using the Kaplan Meier-plotter website revealed that low-level SORBS1 expression (in specimens obtained from patients who were treated with chemotherapy) positively correlated with lower OS among breast cancer or lung cancer patients. Considered together, these results suggest a negative role for SORBS1 in responsivity to cancer chemotherapy.

Previous work has shown that (in some cases) JNK is a major regulator of cisplatin-induced apoptosis in cancer cells [44–46]. However, the work described here suggests that the role of SORBS1 in drug resistance might be independent of JNK activation. Notably, we demonstrated that SORBS1 may stimulate cisplatin sensitivity via increase of p53 protein levels in various cancer cell lines. However, the exact mechanism(s) whereby SORBS1 upregulates p53 level during chemotherapy remains unclear and will be the subject of further research in our laboratory. Given the promotion of SORBS1 in anti-cancer-drug sensitivity, we are planning to initiate a computational analysis and screening program to identify compounds that bind SORBS1. The resulting compound would then be used for the design of structural analogues that could serve as agonists of SORBS1 that permit the reactivation of the p53 pathway to counter chemo-resistance. In the short term, SORBS1 is expected to serve as a valuable marker for screening clinical specimens for metastatic and drug-resistance potential.

In conclusion, our data illustrate for the first time that SORBS1 serves as a potential suppressor of tumorigenesis and metastasis by impeding JNK activation in both breast and lung cancers. Notably, our results provide a novel perspective on the role of the SORBS1 protein in attenuating the efficacy of cisplatin chemotherapy by increasing the accumulation of p53 protein during the treatment of breast and lung cancers. Thus, our studies may serve to explain some of the failures of cisplatin-class drugs in clinical settings; these challenges may, at least in part, reflect deficiencies in SORBS1 (and thus of p53) following chemotherapy treatment. Thus, our results define SORBS1 as a promising therapeutic target for multiple types of cancer.

## MATERIALS AND METHODS

### Cell culture

SUM159, HBL100, MCF7, MDA-MB-468, MDA-MB-435, MDA-MB-231, SKBR3, BT549, and 293T cells were obtained from the American Type Culture Collection (ATCC; Manassas, VA). All of the above cells grown in DMEM medium (Life Technologies) supplemented with 10% fetal bovine serum (FBS; Hyclone Logan, UT, USA) and 1% penicillin/streptomycin (Life Technologies). A549, CRL-1848^TM^, CRL-5803, CRL-5866, CRL-5833, 827, PC-9, 95D, HTB-182, and HTB-177 cells were obtained from the ATCC and maintained in RPMI-1640 (Hyclone) medium. The normal breast epithelial cell line MCF10A was purchased from ATCC and cultured in DMEM/F12 medium (Life Technologies) supplemented with 5% horse serum (Life Technologies), 10 μg/mL insulin (Sigma), 100 ng/mL cholera toxin (Sigma), 20 ng/mL epidermal growth factor (EGF, R&D), 0.5 μg/mL hydrocortisone (Sigma), and 1% penicillin/streptomycin. The normal lung epithelial cell line BEAS-2B was obtained from ATCC and maintained in LHC-8 (Gibco, USA). All cell lines were grown by incubation at 37°C in a humidified 5% CO_2_ atmosphere.

### Plasmids and transfection

The cDNA ORF encoding human SORBS1 was amplified from human genomic DNA by PCR and subcoloned between the EcoRI and XhoI sites of pCMV-C-Myc (Beyotime). Mouse *Sorbs1* was a gift from Dr. Alan Saltiel (Michigan University, USA). Cells were transiently transfected by using Lipofectamine 2000 reagent (Life Technologies) according to the manufacturer's instructions. The transfected cells were incubated for 48 hours at 37°C followed by biochemical and biological assays.

### Western blots

Total cell lysates were prepared in RIPA buffer (Cell Signaling) with complete protease inhibitor cocktail (Roche), phosphatase inhibitors (Roche), 5 mM dithiothreitol (DTT, Sigma) and 1 mM PMSF (Sigma) for 30 min on ice, and then centrifuged at 12,000 × g for 10 minutes at 4°C. The supernatant was recovered and protein concentration was determined by the Bio-Rad protein assay kit (Hercules, CA) according to the manufacturer's instructions. Western blot assays were performed as previously described [13]. The primary antibodies were obtained as follows: anti-SORBS1, anti-GAPDH, and anti-β-actin antibody from Abmart; anti-Hsp90, anti-Snail, anti-Slug, anti-vimentin, anti-JNK, anti-p-JNK, anti-c-Jun, anti-p-c-Jun, anti-p53, anti-caspase3, and anti-cleaved-caspase3 antibodies and horseradish peroxidase (HRP) -conjugated secondary antibodies from Cell Signaling Technology; and anti-E-cadherin and anti-N-cadherin antibodies from BD Transduction Laboratories.

### Vectors and lentivirus transduction

The *SORBS1* short-hairpin RNAi was synthesized and subcloned between the AgeI and EcoRI restriction sites (underlined below) of the pLKO.1 puro lentivirus expression vector (Addgene). The sequences were as follows:

shSORBS1-1 sense : GCAGCAATGGGCAAGACAAAG

shSORBS1-1 antisense: CTTTGTCTTGCCCATTGCTGC

shSORBS1-2 sense : GCAGATGAGTGGAGGCTTTCT

shSORBS1-2 antisense: AGAAAGCCTCCACTCATCTGC

Lentivirus preparation, infection, and selection were performed as described previously [47]. Lentivirus production and transduction were performed according to the vector's technical manual.

### MTT assay

Cells were seeded at suitable densities (depending on the specific experimental procedure) in 96-well plates. After the addition of 20 μL/well of MTT solution (Thiazolyl Blue Tetrazolium Bromide, Sigma, St. Louis, USA), plates were incubated for an additional 6 hr, at which time culture medium was removed from the wells. Dimethyl sulfoxide (DMSO, Sigma, St. Louis, USA) was added at 100 μL/well and plates were incubated for 20 min in a 37°C incubator. Absorbance at a wavelength of 570 nm was measured with a microplate reader (Bio-Rad, CA, USA). For the cell proliferation assay, cell were plated at 5000 cells/well in multiple 96-well plates, and MTT then was added to separate plates after 12, 24, 48, or 72 hr of growth. For the measurement of IC50, cells were seeded at 1 × 10^4^ cells/well. On the following day, medium containing various concentrations of SP600125 (Sigma, St. Louis, USA) or no inhibitor was added to the wells and the plates were incubated for a further 24 hr. Each treatment was performed in four replicate wells. Each experiment was repeated three separate times.

### RNA Isolation and real-time PCR

Total RNA was isolated using Trizol reagent (Invitrogen). Aliquots (1 μg each) of RNA were reverse transcribed using a Takara PrimeScript RT Reagent Kit (Takara) to generate cDNA; reverse transcription was performed according to the manufacturer's instructions. The resulting cDNA was used for real-time PCR with a SYBR^®^ Premix Ex Taq™ Kit (Takara) in a StepOne Real-Time PCR Detection System (Bio-Rad). Values were normalized against those of a housekeeping gene (*GAPDH*). The primers used for real-time PCR are shown as follows:

*SORBS1* F: 5′-ATTCCCAAGCCTTTCCATCAG-3′,

*SORBS1* R: 5′-TTTTGCTGTTCTCGATTGTGTTG-3′,

*E-cadherin* F: 5′-GCCCTGCCAATCCCGATGAAA-3′,

*E-cadherin* R: 5′-GGGGTCAGTATCAGCCGCT-3′,

*N-cadherin* F: 5′-TCAGGCGTCTGTAGAGGCTT-3′,

*N-cadherin* R: 5′-ATGCACATCCTTCGATAAGACTG-3′,

*Vimentin* F: 5′-GCTTCAGAGAGAGGAAGCCGAAAA-3′,

*Vimentin* R: 5′-CCGTGAGGTCAGGCTTGGAAA-3′,

*Slug* F: 5′-CCCAATGGCCTCTCTCCTCTTT-3′,

*Slug* R: 5′-CATCGCAGTGCAGCTGCTTAT-3′,

*Snail* F: 5′-CCAGACCCACTCAGATGTCAAGA A -3′,

*Snail* R: 5′-GGCAGAGGACACAGAACCAGA-3′,

*Zeb1* F: 5′-GATGATGAATGCGAGTCAGATGC-3′,

*Zeb1* R: 5′-ACAGCAGTGTCTTGTTGTTGT-3′,

*Zeb2* F: 5′-CAAGAGGCGCAAACAAGCC-3′,

*Zeb2* R: 5′-GGTTGGCAATACCGTCATCC-3′,

*GAPDH* F: 5′-GGGGAGCCAAAAGGGTCATCATCT-3′,

*GAPDH* R: 5′-GACGCCTGCTTCACCACCTTCTTG-3′,

### Wound-healing assays

Cells were seeded in a 6-well flat-bottomed plate at a density of 4sit^5^/well per the manufacturer's recommendation. Cells were allowed to grow to 90% confluence and then serum starved for 24 hr in medium lacking FBS. After aspiration of the medium, a 200-μL pipette tip was used to scratch each monolayer of cells. Wells were flooded with phosphate-buffered saline (PBS) and emptied to remove detached cells; complete medium then was added and plates were incubated for the indicated time intervals. The scratched cells were photographed at different times as indicated using microscopy. ImageJ software was used to measure cell migration ability by calculating open wound area as a percentages of the initial blank area (containing no cells). Data are presented as the means of three independent experiments.

### Transwell migration and invasion assays

Migration assay was performed in 24-well inserts with 8-μm pores (Corning Inc., Corning, NY, USA), and the cell invasion assay was performed in 24-well inserts with 8-μm pores coated with Matrigel (Corning Inc., Corning, NY, USA) according to manufacturer's protocols. Cells (4 × 10^4^ cells in 200 uL of blank (no FBS) medium containing 0.2% BSA per insert) were seeded in the upper side of each insert and complete medium was added to the bottom side of each insert. After incubation for 24 hr. (for migration assay) or 48 hr (for invasion assay), non-migrated cells were removed from the upper side of the insert with a cotton swab; this process left the migrated cells on the bottom side of the insert. Inserts from both the migration assay and invasion assay were fixed with 4% paraformaldehyde for 20 min, stained with a 0.1% crystal violet solution for 30 min, and photographed using an Olympus IX51 microscope. The numbers of cells were counted using ImageJ software. Data are presented as the means from at least three randomly selected fields.

### 3D cell culture and FLP formation

MCF10A, HBL100, and MDA-MB-231 cells were cultured on Matrigel as described previously [47]. As indicated, cells were propagated for 24 hr (for FLP formation) or 5–12 days (for normal culture). Phase images were captured using an Olympus IX51 microscope.

### Immunofluorescence and confocal imaging

Cells were plated on coverslips or in chamber slides. After growing to a suitable density, cells were fixed with 4% paraformaldehyde, permeabilized in 0.5% Triton X-100, blocked with PBS containing 10% goat serum, and incubated overnight with antibodies at 4°C. Fixed and stained coverslips or slides were washed 3 times with PBS, and primary antibodies were labeled by incubation with Alexa Fluor-conjugated secondary antibodies (Invitrogen). The coverslips or slides then were mounted with mounting medium containing DAPI (Prolong Gold Antifade Reagent, Life Technologies), and cells were visualized with confocal laser microscopy (Carl Zeiss).

### Xenograft tumor model and histological analysis

All animal experimental procedures and protocols were approved by the Institutional Animal Care and Use Committee of the Institute for Nutritional Sciences, Shanghai Institutes for Biological Sciences, Chinese Academy of Science (Shanghai, China). These experiments used either or two stably transfected cell lines (MDA-MB-231 shSORBS1-1 and MDA-MB-231 shSORBS1-2) or a control cell line (MDA-MB-231 Control). Actively growing cells (4 × 10^5^ cells in 100 uL PBS per mouse) were implanted into 6-week-old male nude mice (10 mice/group) by intravenous injection via the tail vein. On Day 21 post-injection, the mice were sacrificed and tissues were harvested for examination by histological analysis.

Specifically, the lungs were fixed with formalin, dehydrated, embedded in paraffin, and sectioned using standard methodologies. Lungs were examined using 6-μm thick, 200-μm step sections stained with haematoxylin and eosin (HE) and evaluated by light microscopy for the presence of micro-metastases. The numbers of micro-metastases were counted in five randomly selected fields under light microscopy in each of five randomly selected tissue sections from each lung. The “average lung metastases” was defined as the calculated mean number of micro-metastases counted by randomly selected four fields under light microscope.

### Cell apoptosis

After growing to 80–90% confluence in 6-well plates, cells were treated with cisplatin as indicated for 24 hr. Apoptotic cells were labeled using the Annexin V apoptosis detection kit (Bio-Vision, SF, USA) according to the manufacturer's instructions, and cells were analyzed using a FACS Canto II flow cytometer (BD Biosciences, USA) and Flowjo software.

### Oncomine data analysis

Datasets from the Oncomine database were analyzed as previously described [48]. Briefly, the gene *SORBS1* was searched using the threshold values as follows: fold-change of 2, *P* value of 0.05, and gene rank in the top 10% among all differentially expressed genes. All the datasets then were listed and ordered by *P* value; for each published dataset, the values were linked to the graphical representations of the original microarray dataset. An unpaired two-tailed Student's *t-*test was used to calculate *P-*value.

### Kaplan meier-plotter analysis

To analyze the prognostic value of SORBS1 in breast cancer or lung cancer, data were analyzed with the KM plotter (http://kmplot.com/analysis/). In brief, for the analyses of the correlaton between SORBS1 level and clinical outcome in breast cancer and lung cancer, the patients who were with or without treatment were selected; and for the analyses of the correlation between SORBS1 level and chemotherapy drug sensitivity, the treated chemotherapy patients were selected. Survival curves were generated by using the Kaplan-Meier Plotter online tool based on data stratified based on the best cut-off. Hazard ratios (HR) and *p* values (log rank *p*) are shown at the top of the panel.

### Statistical analysis

All data are presented as the mean ± s.d. from at least three independent experiments. Two-tailed Student's *t-*tests were conducted using GraphPad 5.0 software. *P* values of less than 0.05 were considered significant differences.

## SUPPLEMENTARY MATERIALS FIGURES AND TABLES




