# Supplementary material for: SORBS1 suppresses tumor metastasis and improves the sensitivity of cancer to chemotherapy drug
Source: Oncotarget. 2016 Oct 24;8(6):9108–22. doi: 10.18632/oncotarget.12851 (PMC5354718; doi:10.18632/oncotarget.12851)
Supplement: Supplementary file 1 [file oncotarget-08-9108-s001.pdf]

SORBS1 suppresses tumor metastasis and improves the sensitivity of cancer to chemotherapy drug

Supplementary Materials

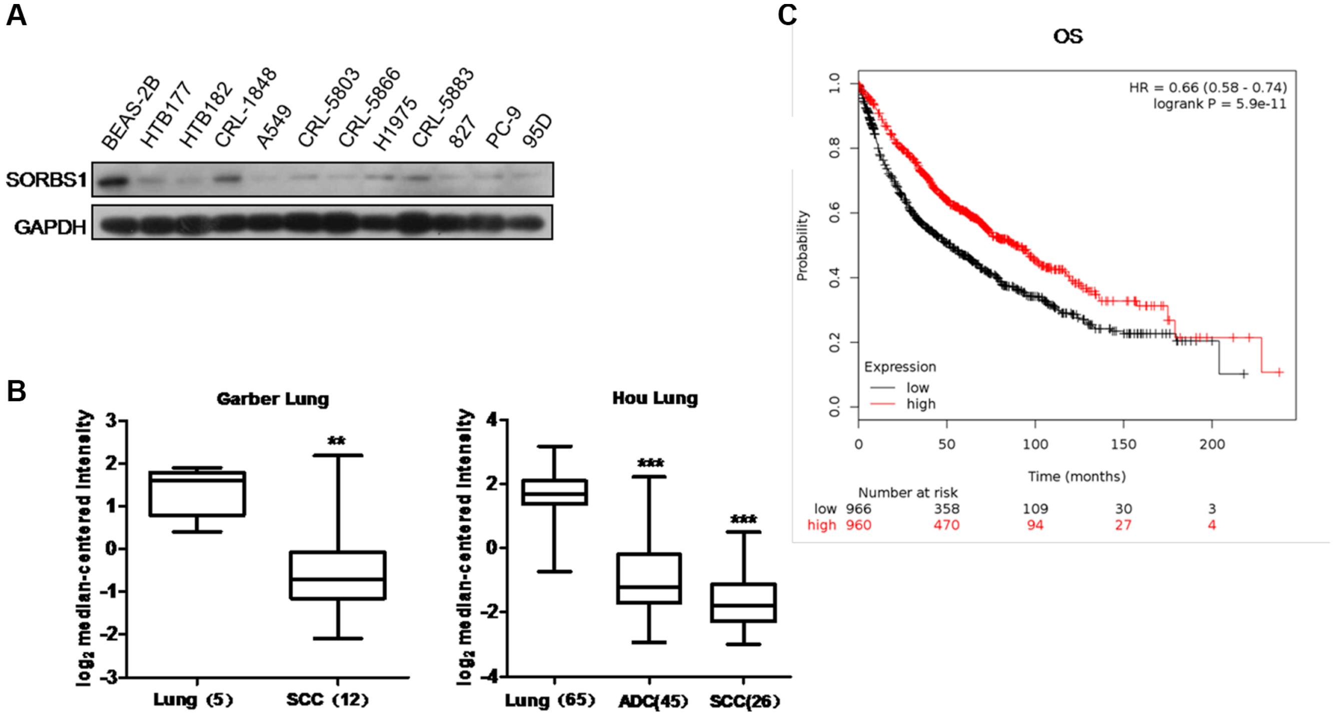

Supplementary Figure S1: SORBS1 is expressed at lower levels in human lung cancer. (**A**) SORBS1 protein levels was detected by western blot in normal lung epithelial cell line 2B and eleven other lung cancer cell lines. (**B**) The comparison of mRNA levels of *SORBS1* between normal lung tissue and squamous cell lung carcinoma (left panel) or lung adenocarcinoma (ADC) in published data sets from oncomine. $^{**}P < 0.01$, $^{***}P < 0.001$. (**C**) Kaplan-Meier survival analysis for assessment of overall survival (OS) of lung cancer based on tumor SORBS1 level in 1926 lung cancer patients. Survival curves were generated by using the Kaplan-Meier Plotter website based on data stratified based on the best cut-off. Curves were compared by hazard ratios (HR) and *p* values (log rank *p*).

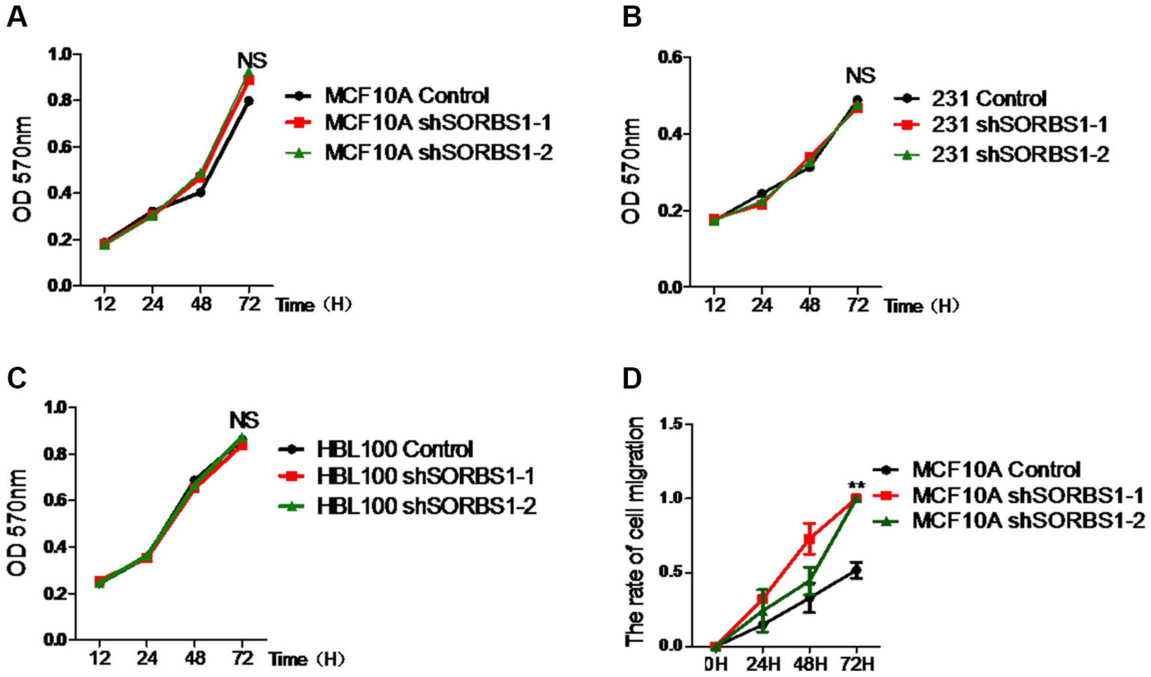

Supplementary Figure S2: Loss of SORBS1 has no effect on cell proliferation but promotes migration in MCF 10A. (**A**–**C**) MTT proliferation assay was performed in MCF 10A (A), MDA-MB-231 (B), HBL100 (C) and their respectively shSORBS1 cell lines. Data represent the mean ± s.d. (n = 3) from three independent expermients. NS: no significance. (**D**) Wound healing assay was conducted at 0, 24, 48 and 72 hours (H) to detect the ability of migration. Data represent the mean ± s.d. (n = 3), $^{**}P < 0.01$, Student's t-test.

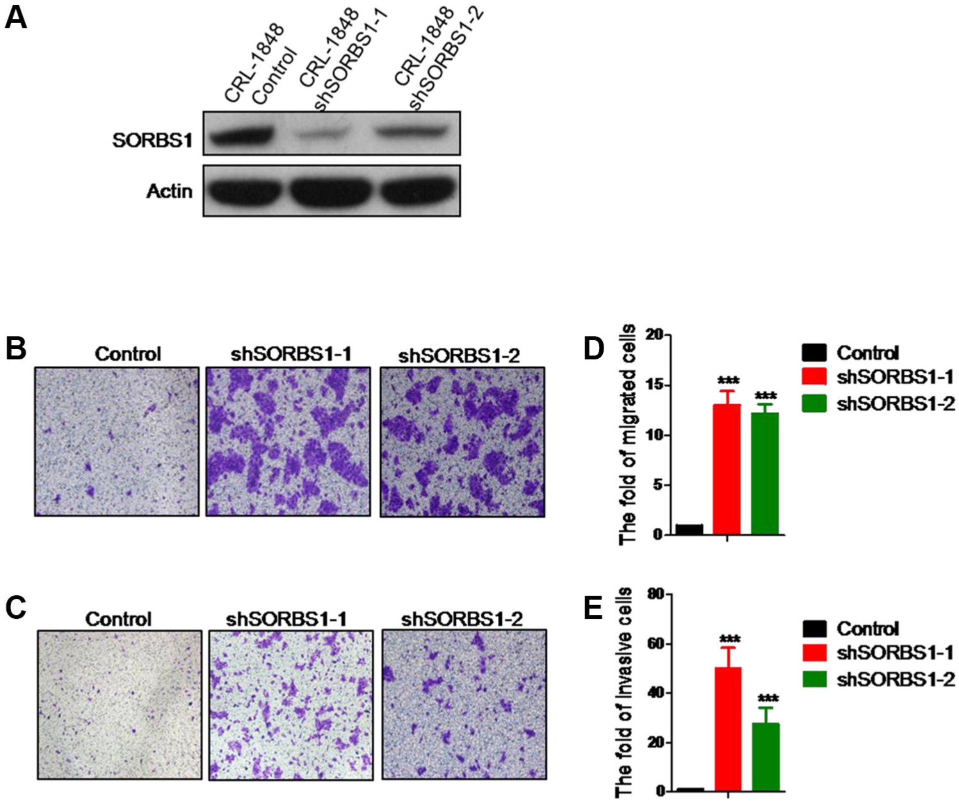

Supplementary Figure S3: Loss of SORBS1 promotes migration and invasion in lung cancer cell. (**A**) Western blot was performed to detect the expression levels of SORBS1 in lung cancer cell CRL-1848™ stably expressing control or shSORBS1. Transwell migration (**B**) and invasion (**C**) assay were conducted in CRL-1848™ control and shSORBS1 cell lines, in which cells (migration, 4×10^4 cells/well; invasion, 8×10^4 cells/well) were cultured for 24 hours. (**D**–**E**) Quantitative results were illustrated for panel B (D) and panel C (E). Data are shown as mean ± s.d. (n = 3), $^{***}P < 0.001$, Student's t-test.

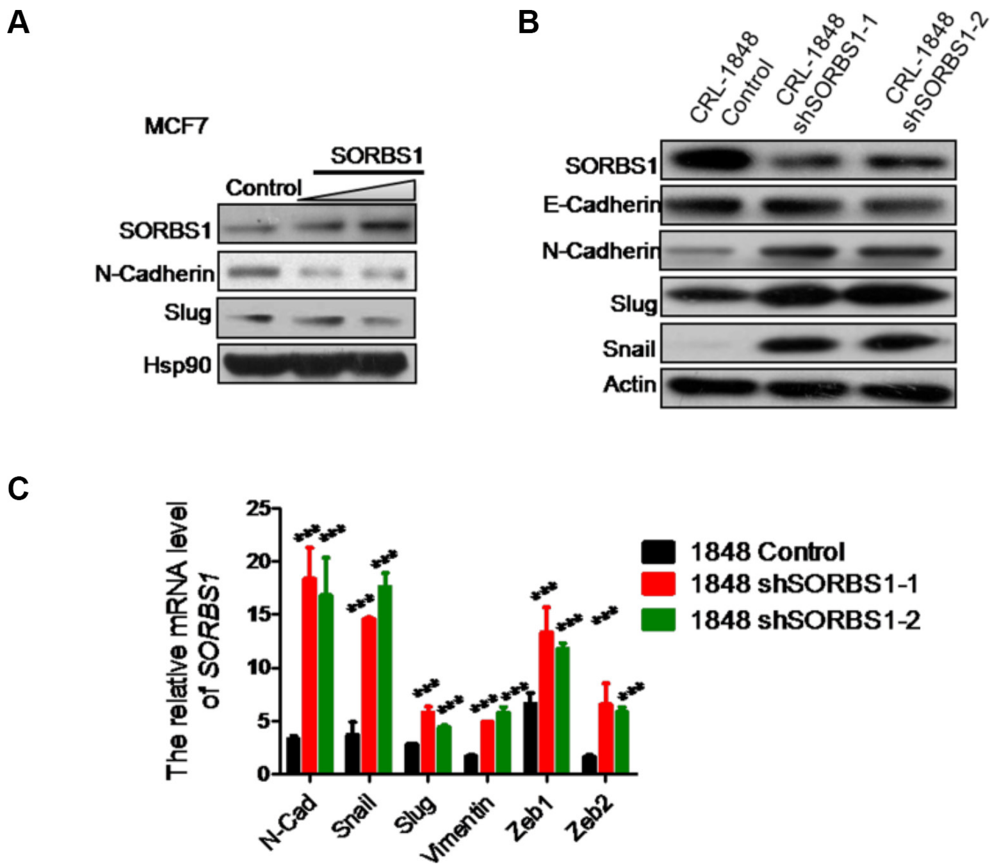

Supplementary Figure S4: SORBS1 inhibits epithelial-to-mesenchymal transition in breast cancer and lung cancer cells. (**A**) MCF7 cells were transiently transfected with control vector, 1 µg or 2 µg human *SORBS1*. After 48 hours, the protein expression of SORBS1 and EMT markers (N-cadherin, Slug) were detected by western blot. (**B**) The levels of SORBS1 and EMT markers were tested by western blot in CRL-1848™ control and shSORBS1 cell lines. (**C**) Quantitative real time PCR was performed to detect the mRNA levels of EMT markers in CRL-1848™ control and shSORBS1 cell lines. Data are shown as mean ± s.d. ($n = 3$), ***$P < 0.001$, Student's *t*-test.

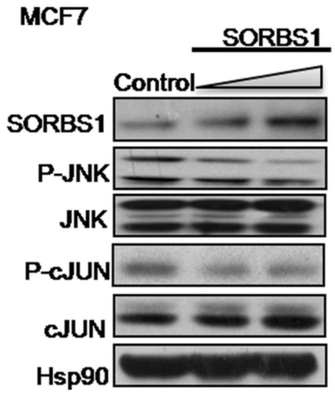

Supplementary Figure S5: Overexpression of SORBS1 inhibits the JNK/c-Jun activation in MCF7. The levels of JNK, P-JNK, c-Jun, P-c-Jun and SORBS1 in MCF7 control and MCF7 SORBS1 cells were detected by western blot.

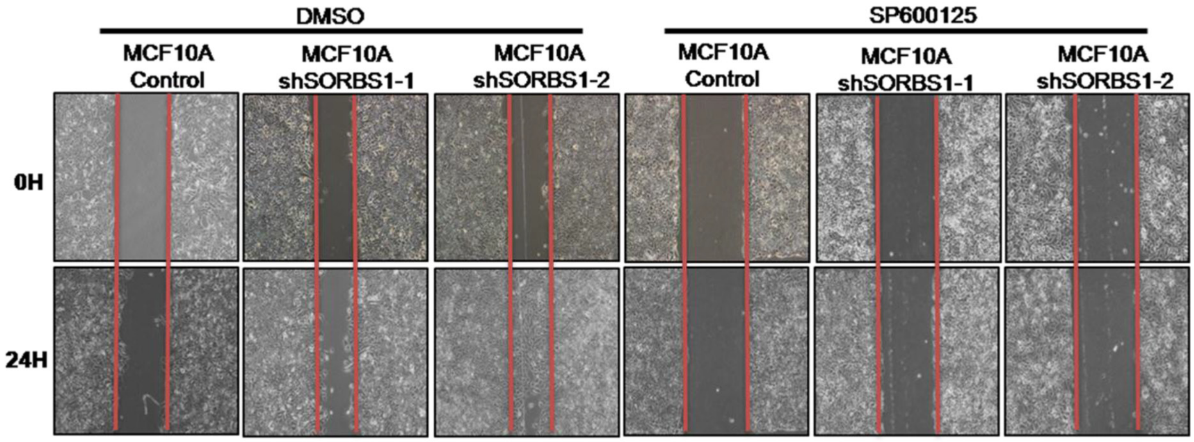

Supplementary Figure S6: Inhibition of JNK activity decreases migration in MCF 10A shSORBS1 cells. Migration were detected by wound healing in MCF 10A control and MCF10A.shSORBS1 cells treated with DMSO or sp600125 (10 μM). Images were taken at 0 and 24 hours (**H**) with a 10× objective lens.

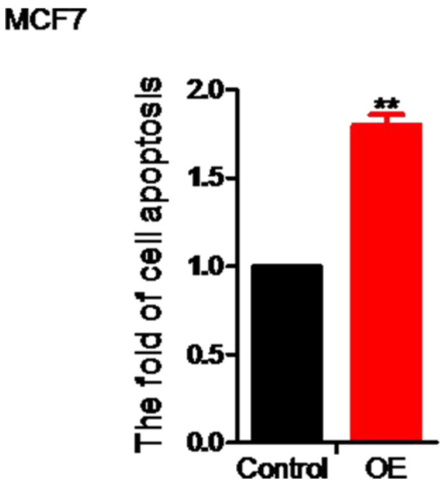

Supplementary Figure S7: SORBS1 increases cisplatin induced-apoptosis in MCF7. MCF7 cells were transiently transfected with 1 μg of vector (Control) or human *SORBS1* (OE). After 24 hours, cells were treated with 10 ug/ml cisplatin for another 24 hours. The rate of apoptosis was analyzed by FCM. Data are shown as mean ± s.d. (n = 3). **P < 0.01, Student's t-test.

Supplementary Table S1: Original values of the data sets in breast cancer VS normal breast obtained from oncomine. See Supplementary_Table_S1.

Supplementary Table S2: Original values of the data sets in invasive breast cancer VS breast cancer obtained from oncomine. See Supplementary_Table_S2.

Supplementary Table S3: Original values of the data sets in lung cancer VS normal lung tissue obtained from oncomine. See Supplementary_Table_S3.
