# Supplementary material for: SORBS1 suppresses tumor metastasis and improves the sensitivity of cancer to chemotherapy drug
Source: Oncotarget. 2016 Oct 24;8(6):9108–22. doi: 10.18632/oncotarget.12851 (PMC5354718; doi:10.18632/oncotarget.12851)
Supplement: Supplementary file 2 [file oncotarget-08-9108-s002.docx]

| **Supplementary Table S1: Original** values **of the data sets in breast cancer VS normal breast obtained from oncomine** | | | | | | | |
| --- | --- | --- | --- | --- | --- | --- | --- |
| Richardson Breast 2 | | Curtis Breast | | | | | |
| Title | Value | Title | Value | Title | Value | Title | Value |
| NB42 U133p2 | 3.300598198 | Normal1 | 3.30264 | Normal54 | 1.32976 | Normal107 | 0.49431 |
| NB58 U133p2 | 3.00929342 | Normal2 | 3.06025 | Normal55 | 1.32581 | Normal108 | 0.46538 |
| NB60 U133p2 | 3.325199301 | Normal3 | 2.97276 | Normal56 | 1.27931 | Normal109 | 0.4619 |
| NB64 U133p2 | 3.075792738 | Normal4 | 2.98439 | Normal57 | 1.25528 | Normal110 | 0.44785 |
| NB69 U133p2 | 3.16933979 | Normal5 | 2.93334 | Normal58 | 1.24809 | Normal111 | 0.43205 |
| NB83 U133p2 | 3.27029271 | Normal6 | 2.6259 | Normal59 | 1.2424 | Normal112 | 0.42721 |
| NB87 U133p2 | 3.124339716 | Normal7 | 2.57181 | Normal60 | 1.23553 | Normal113 | 0.39646 |
| T118 U133p2 | 2.035088958 | Normal8 | 2.57087 | Normal61 | 1.20498 | Normal114 | 0.39185 |
| T134 U133p2 | 2.684670646 | Normal9 | 2.53385 | Normal62 | 1.18837 | Normal115 | 0.38119 |
| T140 U133p2 | 1.835637123 | Normal10 | 2.44653 | Normal63 | 1.16191 | Normal116 | 0.36185 |
| T141 U133p2 | 2.805808349 | Normal11 | 2.4033 | Normal64 | 1.14341 | Normal117 | 0.34464 |
| T146 U133p2 | 2.773281128 | Normal12 | 2.36468 | Normal65 | 1.1121 | Normal118 | 0.34274 |
| T147 U133p2 | 2.912274294 | Normal13 | 2.3555 | Normal66 | 1.07983 | Normal119 | 0.34077 |
| T149 U133p2 | 2.202759351 | Normal14 | 2.31523 | Normal67 | 1.07339 | Normal120 | 0.33679 |
| T133 U133p2 | 1.919511708 | Normal15 | 2.26125 | Normal68 | 1.05895 | Normal121 | 0.33557 |
| T21 U133p2 | 3.006477564 | Normal16 | 2.23361 | Normal69 | 1.03908 | Normal122 | 0.32647 |
| T56 U133p2 | 2.44662149 | Normal17 | 2.22839 | Normal70 | 1.02218 | Normal123 | 0.31829 |
| T116 U133p2 | 2.480699416 | Normal18 | 2.19214 | Normal71 | 1.00846 | Normal124 | 0.31088 |
| T144 U133p2 | 2.386118397 | Normal19 | 2.17325 | Normal72 | 0.9934 | Normal125 | 0.30364 |
| T129 U133p2 | 2.534101455 | Normal20 | 2.16581 | Normal73 | 0.98735 | Normal126 | 0.28892 |
| T143 U133p2 | 2.119867421 | Normal21 | 2.16366 | Normal74 | 0.97175 | Normal127 | 0.28338 |
| T38 U133p2 | 2.26797618 | Normal22 | 2.13243 | Normal75 | 0.9532 | Normal128 | 0.26979 |
| T123 U133p2 | 2.147508597 | Normal23 | 2.08283 | Normal76 | 0.94804 | Normal129 | 0.26118 |
| T137 U133p2 | 2.583196523 | Normal24 | 2.06313 | Normal77 | 0.92338 | Normal130 | 0.25282 |
| T130 U133p2 | 1.814882605 | Normal25 | 2.06179 | Normal78 | 0.91655 | Normal131 | 0.22094 |
| T151 U133p2 | 1.952102418 | Normal26 | 2.02594 | Normal79 | 0.91495 | Normal132 | 0.22085 |
| T152 U133p2 | 3.097833251 | Normal27 | 1.98075 | Normal80 | 0.90004 | Normal133 | 0.21994 |
| T37 U133p2 | 2.416266873 | Normal28 | 1.96468 | Normal81 | 0.86267 | Normal134 | 0.15136 |
| T183 U133p2 | 1.9801862 | Normal29 | 1.94242 | Normal82 | 0.85675 | Normal135 | 0.05463 |
| T117 U133p2 | 2.243864027 | Normal30 | 1.89723 | Normal83 | 0.81748 | Normal136 | −0.17691 |
| T161 U133p2 | 2.374574913 | Normal31 | 1.88914 | Normal84 | 0.81431 | Normal137 | −0.28301 |
| T30 U133p2 | 2.994329901 | Normal32 | 1.87723 | Normal85 | 0.76785 | Normal138 | −0.01781 |
| T84 U133p2 | 2.445639313 | Normal33 | 1.86741 | Normal86 | 0.73204 | Normal139 | 0.03768 |
| T115 U133p2 | 1.958946583 | Normal34 | 1.84096 | Normal87 | 0.70655 | Normal140 | 0.02391 |
| T44 U133p2 | 2.792309815 | Normal35 | 1.82603 | Normal88 | 0.70446 | Normal141 | 0.01439 |
| T81 U133p2 | 2.602226437 | Normal36 | 1.73339 | Normal89 | 0.69637 | Normal142 | 0.01068 |
| T50 U133p2 | 2.796471087 | Normal37 | 1.65393 | Normal90 | 0.69565 | Normal143 | 0.00724 |
| T4 U133p2 | 2.69087073 | Normal38 | 1.60105 | Normal91 | 0.68906 | Normal144 | 0.00152 |
| T175 U133p2 | 1.993116376 | Normal39 | 1.55426 | Normal92 | 0.66018 | Cancer1 | −0.30786 |
| T178 U133p2 | 2.485295433 | Normal40 | 1.55371 | Normal93 | 0.65563 | Cancer2 | −0.25822 |
| T41 U133p2 | 2.896002469 | Normal41 | 1.50442 | Normal94 | 0.64227 | Cancer3 | −0.23189 |
| T73 U133p2 | 2.737280067 | Normal42 | 1.50252 | Normal95 | 0.63405 | Cancer4 | −0.08325 |
| T92 U133p2 | 2.536545322 | Normal43 | 1.48924 | Normal96 | 0.62553 | Cancer5 | −0.06619 |
| T74 U133p2 | 2.759486103 | Normal44 | 1.48893 | Normal97 | 0.62065 | Cancer6 | −0.05339 |
| T162 U133p2 | 2.355836641 | Normal45 | 1.47026 | Normal98 | 0.61159 | Cancer7 | −0.0243 |
| T145 U133p2 | 1.856093313 | Normal46 | 1.46683 | Normal99 | 0.59014 | Cancer8 | 0.03009 |
| T119 U133p2 | 2.424322856 | Normal47 | 1.43856 | Normal100 | 0.58513 | Cancer9 | 0.04855 |
|  |  | Normal48 | 1.43652 | Normal101 | 0.58512 | Cancer10 | 0.07436 |
|  |  | Normal49 | 1.39097 | Normal102 | 0.54892 | Cancer11 | 0.10893 |
|  |  | Normal50 | 1.38839 | Normal103 | 0.53507 | Cancer12 | 0.47966 |
|  |  | Normal51 | 1.37433 | Normal104 | 0.52962 | Cancer13 | 0.33122 |
|  |  | Normal52 | 1.36609 | Normal105 | 0.5241 | Cancer14 | 0.00793 |
|  |  | Normal53 | 1.33715 | Normal106 | 0.50175 |  |  |

| **Supplementary Table S2: Original** values **of the data sets in invasive breast cancer VS breast cancer obtained from Oncomine** | | | | | |
| --- | --- | --- | --- | --- | --- |
| Nikolsky Breast | | | | | |
| Title | Value | Title | Value | Title | Value |
| Ductal breast carcinoma1 | −0.0301 | Invasive Ductal breast carcinoma 42 | −0.0317 | Invasive Ductal breast carcinoma 88 | 0.0438 |
| Ductal breast carcinoma2 | 0.0139 | Invasive Ductal breast carcinoma 43 | −0.0169 | Invasive Ductal breast carcinoma 89 | 0.0459 |
| Ductal breast carcinoma3 | 0.1568 | Invasive Ductal breast carcinoma 44 | −0.0158 | Invasive Ductal breast carcinoma 90 | 0.0491 |
| Ductal breast carcinoma4 | 0.2057 | Invasive Ductal breast carcinoma 45 | −0.0155 | Invasive Ductal breast carcinoma 91 | 0.0513 |
| Ductal breast carcinoma5 | 0.2874 | Invasive Ductal breast carcinoma 46 | −0.0115 | Invasive Ductal breast carcinoma 92 | 0.0521 |
| Invasive Ductal breast carcinoma 1 | −0.6646 | Invasive Ductal breast carcinoma 47 | −0.0078 | Invasive Ductal breast carcinoma 93 | 0.0522 |
| Invasive Ductal breast carcinoma 2 | −0.4681 | Invasive Ductal breast carcinoma 48 | −0.00764 | Invasive Ductal breast carcinoma 94 | 0.054 |
| Invasive Ductal breast carcinoma 3 | −0.4396 | Invasive Ductal breast carcinoma 49 | −0.0064 | Invasive Ductal breast carcinoma 95 | 0.0545 |
| Invasive Ductal breast carcinoma 4 | −0.4107 | Invasive Ductal breast carcinoma 50 | −0.0042 | Invasive Ductal breast carcinoma 96 | 0.0588 |
| Invasive Ductal breast carcinoma 5 | −0.352 | Invasive Ductal breast carcinoma 51 | −0.0035 | Invasive Ductal breast carcinoma 97 | 0.0601 |
| Invasive Ductal breast carcinoma 6 | −0.3386 | Invasive Ductal breast carcinoma 52 | −0.0022 | Invasive Ductal breast carcinoma 98 | 0.0604 |
| Invasive Ductal breast carcinoma 7 | −0.318 | Invasive Ductal breast carcinoma 53 | −0.0018 | Invasive Ductal breast carcinoma 99 | 0.0615 |
| Invasive Ductal breast carcinoma 8 | −0.285 | Invasive Ductal breast carcinoma 54 | −0.0016 | Invasive Ductal breast carcinoma 100 | 0.0641 |
| Invasive Ductal breast carcinoma 9 | −0.2842 | Invasive Ductal breast carcinoma 55 | −0.0014 | Invasive Ductal breast carcinoma 101 | 0.0673 |
| Invasive Ductal breast carcinoma 10 | −0.2689 | Invasive Ductal breast carcinoma 56 | −0.0013 | Invasive Ductal breast carcinoma 102 | 0.0683 |
| Invasive Ductal breast carcinoma 11 | −0.2569 | Invasive Ductal breast carcinoma 57 | −0.0001 | Invasive Ductal breast carcinoma 103 | 0.0689 |
| Invasive Ductal breast carcinoma 12 | −0.2327 | Invasive Ductal breast carcinoma 58 | 0.00002 | Invasive Ductal breast carcinoma 104 | 0.0719 |
| Invasive Ductal breast carcinoma 13 | −0.2266 | Invasive Ductal breast carcinoma 59 | 0.0013 | Invasive Ductal breast carcinoma 105 | 0.0724 |
| Invasive Ductal breast carcinoma 14 | −0.225 | Invasive Ductal breast carcinoma 60 | 0.0042 | Invasive Ductal breast carcinoma 106 | 0.076 |
| Invasive Ductal breast carcinoma 15 | −0.2066 | Invasive Ductal breast carcinoma 61 | 0.0044 | Invasive Ductal breast carcinoma 107 | 0.0777 |
| Invasive Ductal breast carcinoma 16 | −0.2059 | Invasive Ductal breast carcinoma 62 | 0.0054 | Invasive Ductal breast carcinoma 108 | 0.0811 |
| Invasive Ductal breast carcinoma 17 | −0.2053 | Invasive Ductal breast carcinoma 63 | 0.0077 | Invasive Ductal breast carcinoma 109 | 0.0819 |
| Invasive Ductal breast carcinoma 18 | −0.1827 | Invasive Ductal breast carcinoma 64 | 0.0085 | Invasive Ductal breast carcinoma 110 | 0.0832 |
| Invasive Ductal breast carcinoma 19 | −0.1611 | Invasive Ductal breast carcinoma 65 | 0.0088 | Invasive Ductal breast carcinoma 111 | 0.0835 |
| Invasive Ductal breast carcinoma 20 | −0.1408 | Invasive Ductal breast carcinoma 66 | 0.009 | Invasive Ductal breast carcinoma 112 | 0.0843 |
| Invasive Ductal breast carcinoma 21 | −0.138 | Invasive Ductal breast carcinoma 67 | 0.0108 | Invasive Ductal breast carcinoma 113 | 0.0852 |
| Invasive Ductal breast carcinoma 22 | −0.1234 | Invasive Ductal breast carcinoma 68 | 0.0161 | Invasive Ductal breast carcinoma 114 | 0.0942 |
| Invasive Ductal breast carcinoma 23 | −0.1195 | Invasive Ductal breast carcinoma 69 | 0.0165 | Invasive Ductal breast carcinoma 115 | 0.0949 |
| Invasive Ductal breast carcinoma 24 | −0.1183 | Invasive Ductal breast carcinoma 70 | 0.0177 | Invasive Ductal breast carcinoma 116 | 0.0989 |
| Invasive Ductal breast carcinoma 25 | −0.1127 | Invasive Ductal breast carcinoma 71 | 0.0185 | Invasive Ductal breast carcinoma 117 | 0.0999 |
| Invasive Ductal breast carcinoma 26 | −0.1108 | Invasive Ductal breast carcinoma 72 | 0.0194 | Invasive Ductal breast carcinoma 118 | 0.1085 |
| Invasive Ductal breast carcinoma 27 | −0.1032 | Invasive Ductal breast carcinoma 73 | 0.0261 | Invasive Ductal breast carcinoma 119 | 0.1222 |
| Invasive Ductal breast carcinoma 28 | −0.0849 | Invasive Ductal breast carcinoma 74 | 0.0271 | Invasive Ductal breast carcinoma 120 | 0.1273 |
| Invasive Ductal breast carcinoma 29 | −0.0763 | Invasive Ductal breast carcinoma 75 | 0.0274 | Invasive Ductal breast carcinoma 121 | 0.1296 |
| Invasive Ductal breast carcinoma 30 | −0.0687 | Invasive Ductal breast carcinoma 76 | 0.0299 | Invasive Ductal breast carcinoma 122 | 0.1318 |
| Invasive Ductal breast carcinoma 31 | −0.0671 | Invasive Ductal breast carcinoma 77 | 0.03 | Invasive Ductal breast carcinoma 123 | 0.1559 |
| Invasive Ductal breast carcinoma 32 | −0.0659 | Invasive Ductal breast carcinoma 78 | 0.0314 | Invasive Ductal breast carcinoma 124 | 0.1676 |
| Invasive Ductal breast carcinoma 33 | −0.0594 | Invasive Ductal breast carcinoma 79 | 0.0324 | Invasive Ductal breast carcinoma 125 | 0.1973 |
| Invasive Ductal breast carcinoma 34 | −0.0587 | Invasive Ductal breast carcinoma 80 | 0.0341 | Invasive Ductal breast carcinoma 126 | 0.2025 |
| Invasive Ductal breast carcinoma 35 | −0.058 | Invasive Ductal breast carcinoma 81 | 0.0344 | Invasive Ductal breast carcinoma 127 | 0.2197 |
| Invasive Ductal breast carcinoma 36 | −0.0514 | Invasive Ductal breast carcinoma 82 | 0.0344 | Invasive Ductal breast carcinoma 128 | 0.2315321 |
| Invasive Ductal breast carcinoma 37 | −0.0415 | Invasive Ductal breast carcinoma 83 | 0.0353 | Invasive Ductal breast carcinoma 129 | 0.2359 |
| Invasive Ductal breast carcinoma 38 | −0.0413 | Invasive Ductal breast carcinoma 84 | 0.0354 | Invasive Ductal breast carcinoma 130 | 0.2366 |
| Invasive Ductal breast carcinoma 39 | −0.0381 | Invasive Ductal breast carcinoma 85 | 0.0365 | Invasive Ductal breast carcinoma 131 | 0.2424 |
| Invasive Ductal breast carcinoma 40 | −0.0378 | Invasive Ductal breast carcinoma 86 | 0.0378 | Invasive Ductal breast carcinoma 132 | 0.257 |
| Invasive Ductal breast carcinoma 41 | −0.0331 | Invasive Ductal breast carcinoma 87 | 0.0396 | Invasive Ductal breast carcinoma 133 | 0.4309 |

| **Curtis Breast** | | | | | | | | | |
| --- | --- | --- | --- | --- | --- | --- | --- | --- | --- |
| **Title** | **Value** | **Title** | **Value** | **Title** | **Value** | **Title** | **Value** | **Title** | **Value** |
| Breast1 | 6.56282 | Breast49 | 5.2899 | Breast97 | 3.57873 | Invasive breast carcinoma1 | 0.462 | Invasive breast carcinoma49 | 1.92157 |
| Breast2 | 6.47659 | Breast50 | 5.24382 | Breast98 | 3.49929 | Invasive breast carcinoma2 | 0.57398 | Invasive breast carcinoma50 | 1.97614 |
| Breast3 | 6.41893 | Breast51 | 5.19774 | Breast99 | 3.49757 | Invasive breast carcinoma3 | 0.62218 | Invasive breast carcinoma51 | 1.98655 |
| Breast4 | 6.41122 | Breast52 | 5.1841 | Breast100 | 3.47538 | Invasive breast carcinoma4 | 0.83538 | Invasive breast carcinoma52 | 1.98962 |
| Breast5 | 6.39133 | Breast53 | 5.17977 | Breast101 | 3.46794 | Invasive breast carcinoma5 | 0.85373 | Invasive breast carcinoma53 | 2.00662 |
| Breast6 | 6.34136 | Breast54 | 5.16255 | Breast102 | 3.46343 | Invasive breast carcinoma6 | 0.87918 | Invasive breast carcinoma54 | 2.05577 |
| Breast7 | 6.30765 | Breast55 | 5.15736 | Breast103 | 3.43917 | Invasive breast carcinoma7 | 0.89755 | Invasive breast carcinoma55 | 2.08647 |
| Breast8 | 6.29699 | Breast56 | 5.02726 | Breast104 | 3.42835 | Invasive breast carcinoma8 | 0.95067 | Invasive breast carcinoma56 | 2.12561 |
| Breast9 | 6.23159 | Breast57 | 5.01605 | Breast105 | 3.41846 | Invasive breast carcinoma9 | 0.95594 | Invasive breast carcinoma57 | 2.14831 |
| Breast10 | 6.21307 | Breast58 | 4.9885 | Breast106 | 3.37436 | Invasive breast carcinoma10 | 0.98969 | Invasive breast carcinoma58 | 2.15047 |
| Breast11 | 6.18727 | Breast59 | 4.92149 | Breast107 | 3.36024 | Invasive breast carcinoma11 | 1.0047 | Invasive breast carcinoma59 | 2.15191 |
| Breast12 | 6.16389 | Breast60 | 4.91036 | Breast108 | 3.32585 | Invasive breast carcinoma12 | 1.03586 | Invasive breast carcinoma60 | 2.15391 |
| Breast13 | 6.14998 | Breast61 | 4.88218 | Breast109 | 3.26412 | Invasive breast carcinoma13 | 1.05095 | Invasive breast carcinoma61 | 2.1559 |
| Breast14 | 6.13455 | Breast62 | 4.872 | Breast110 | 3.22556 | Invasive breast carcinoma14 | 1.05639 | Invasive breast carcinoma62 | 2.19047 |
| Breast15 | 6.11618 | Breast63 | 4.85301 | Breast111 | 3.2244 | Invasive breast carcinoma15 | 1.08406 | Invasive breast carcinoma63 | 2.19428 |
| Breast16 | 6.07783 | Breast64 | 4.83402 | Breast112 | 3.20531 | Invasive breast carcinoma16 | 1.10931 | Invasive breast carcinoma64 | 2.20453 |
| Breast17 | 6.03417 | Breast65 | 4.82532 | Breast113 | 3.20324 | Invasive breast carcinoma17 | 1.14948 | Invasive breast carcinoma65 | 2.21608 |
| Breast18 | 6.02198 | Breast66 | 4.81576 | Breast114 | 3.17658 | Invasive breast carcinoma18 | 1.16274 | Invasive breast carcinoma66 | 2.22027 |
| Breast19 | 6.02188 | Breast67 | 4.75255 | Breast115 | 3.14144 | Invasive breast carcinoma19 | 1.16558 | Invasive breast carcinoma67 | 2.26653 |
| Breast20 | 6.00969 | Breast68 | 4.73765 | Breast116 | 3.00513 | Invasive breast carcinoma20 | 1.19778 | Invasive breast carcinoma68 | 2.27194 |
| Breast21 | 5.96669 | Breast69 | 4.67908 | Breast117 | 2.99663 | Invasive breast carcinoma21 | 1.23907 | Invasive breast carcinoma69 | 2.29709 |
| Breast22 | 5.94738 | Breast70 | 4.64236 | Breast118 | 2.95895 | Invasive breast carcinoma22 | 1.24776 | Invasive breast carcinoma70 | 2.41899 |
| Breast23 | 5.94516 | Breast71 | 4.61672 | Breast119 | 2.95876 | Invasive breast carcinoma23 | 1.27408 | Invasive breast carcinoma71 | 2.43897 |
| Breast24 | 5.91815 | Breast72 | 4.60874 | Breast120 | 2.94756 | Invasive breast carcinoma24 | 1.29778 | Invasive breast carcinoma72 | 2.44411 |
| Breast25 | 5.90039 | Breast73 | 4.59505 | Breast121 | 2.93144 | Invasive breast carcinoma25 | 1.34342 | Invasive breast carcinoma73 | 2.46438 |
| Breast26 | 5.89954 | Breast74 | 4.53103 | Breast122 | 2.91617 | Invasive breast carcinoma26 | 1.35605 | Invasive breast carcinoma74 | 2.60322 |
| Breast27 | 5.85415 | Breast75 | 4.5304 | Breast123 | 2.90942 | Invasive breast carcinoma27 | 1.37462 | Invasive breast carcinoma75 | 2.61439 |
| Breast28 | 5.82121 | Breast76 | 4.52436 | Breast124 | 2.90655 | Invasive breast carcinoma28 | 1.38537 | Invasive breast carcinoma76 | 2.66466 |
| Breast29 | 5.81259 | Breast77 | 4.49934 | Breast125 | 2.8769 | Invasive breast carcinoma29 | 1.42047 | Invasive breast carcinoma77 | 2.68547 |
| Breast30 | 5.70036 | Breast78 | 4.45916 | Breast126 | 2.78292 | Invasive breast carcinoma30 | 1.44347 | Invasive breast carcinoma78 | 2.75708 |
| Breast31 | 5.69708 | Breast79 | 4.42388 | Breast127 | 2.75494 | Invasive breast carcinoma31 | 1.47174 | Invasive breast carcinoma79 | 2.7558 |
| Breast32 | 5.68718 | Breast80 | 4.27027 | Breast128 | 2.73248 | Invasive breast carcinoma32 | 1.49393 | Invasive breast carcinoma80 | 2.81651 |
| Breast33 | 5.67718 | Breast81 | 4.2551 | Breast129 | 2.72821 | Invasive breast carcinoma33 | 1.50579 | Invasive breast carcinoma81 | 2.8412 |
| Breast34 | 5.67412 | Breast82 | 4.2106 | Breast130 | 2.67271 | Invasive breast carcinoma34 | 1.50702 | Invasive breast carcinoma82 | 2.84672 |
| Breast35 | 5.60349 | Breast83 | 4.1866 | Breast131 | 2.62617 | Invasive breast carcinoma35 | 1.53458 | Invasive breast carcinoma83 | 2.96091 |
| Breast36 | 5.52974 | Breast84 | 4.14636 | Breast132 | 2.61861 | Invasive breast carcinoma36 | 1.55911 | Invasive breast carcinoma84 | 3.09094 |
| Breast37 | 5.5139 | Breast85 | 4.06035 | Breast133 | 2.59763 | Invasive breast carcinoma37 | 1.57016 | Invasive breast carcinoma85 | 3.20382 |
| Breast38 | 5.45548 | Breast86 | 4.05171 | Breast134 | 2.58884 | Invasive breast carcinoma38 | 1.61772 | Invasive breast carcinoma86 | 3.61846 |
| Breast39 | 5.43821 | Breast87 | 4.04963 | Breast135 | 2.5458 | Invasive breast carcinoma39 | 1.63595 | Invasive breast carcinoma87 | 3.66862 |
| Breast40 | 5.41235 | Breast88 | 4.04689 | Breast136 | 2.5126 | Invasive breast carcinoma40 | 1.64386 | Invasive breast carcinoma88 | 3.77878 |
| Breast41 | 5.40441 | Breast89 | 4.044 | Breast137 | 2.30771 | Invasive breast carcinoma41 | 1.66859 | Invasive breast carcinoma89 | 3.8576 |
| Breast42 | 5.37821 | Breast90 | 3.85535 | Breast138 | 2.29366 | Invasive breast carcinoma42 | 1.75717 | Invasive breast carcinoma90 | 5.68583 |
| Breast43 | 5.37492 | Breast91 | 3.85362 | Breast139 | 2.28587 | Invasive breast carcinoma43 | 1.75823 |  |  |
| Breast44 | 5.34341 | Breast92 | 3.84067 | Breast140 | 2.2792 | Invasive breast carcinoma44 | 1.76091 |  |  |
| Breast45 | 5.31592 | Breast93 | 3.807 | Breast141 | 2.24731 | Invasive breast carcinoma45 | 1.76952 |  |  |
| Breast46 | 5.30877 | Breast94 | 3.73296 | Breast142 | 2.13122 | Invasive breast carcinoma46 | 1.79463 |  |  |
| Breast47 | 5.30253 | Breast95 | 3.65145 | Breast143 | 2.13107 | Invasive breast carcinoma47 | 1.8336 |  |  |
| Breast48 | 5.30108 | Breast96 | 3.64261 | Breast144 | 1.75669 | Invasive breast carcinoma48 | 1.87554 |  |  |

| **Supplementary Table S3: Original** values **of the data sets in Lung cancer VS normal lung tissue obtained from Oncomine** | | | | | | | | | |
| --- | --- | --- | --- | --- | --- | --- | --- | --- | --- |
| Garber Lung | | Hou Lung | | | | | | | |
| Title | Value | Title | Value | Title | Value | Title | Value | Title | Value |
| Normal1 | 0.4 | NSCLC EMC 2333N | 1.76125 | NSCLC EMC LCC2334N | 2.37356 | NSCLC EMC 2420T | −2.8128 | NSCLC EMC TB0041tu | −0.803498 |
| Normal2 | 1.607 | NSCLC EMC 2335N | 2.08664 | NSCLC EMC SCC2213N | 2.06696 | NSCLC EMC 2439T | 2.2133 | NSCLC EMC TB0044tu | −0.217833 |
| Normal3 | 1.173 | NSCLC EMC 2344T | 2.43599 | NSCLC EMC SCC2235N | 1.50672 | NSCLC EMC ADC2243T | −0.0800761 | NSCLC EMC TB0045tu | 0.0801291 |
| Normal4 | 1.677 | NSCLC EMC 2381N | 1.58261 | NSCLC EMC SCC2239N | 2.07654 | NSCLC EMC ADC2246T | 0.622198 | NSCLC EMC TB0047tu | −0.314284 |
| Normal5 | 1.899 | NSCLC EMC 2387N | 1.91091 | NSCLC EMC SCC2261N | 1.453 | NSCLC EMC ADC2248T | −0.736002 | NSCLC EMC TB0052tu | −1.39239 |
| SSC1 | −2.092 | NSCLC EMC 2393N | 2.03389 | NSCLC EMC SCC2265N | 0.752617 | NSCLC EMC ADC2256T | −1.16668 | NSCLC EMC TB0062tu | −2.27607 |
| SSC2 | −1.589 | NSCLC EMC 2394T | −0.738717 | NSCLC EMC SCC2267N | 1.58936 | NSCLC EMC ADC2260T | −0.669343 | NSCLC EMC TB0067tu | −1.68019 |
| SSC3 | −1.178 | NSCLC EMC 2405N | 2.57482 | NSCLC EMC SCC2273N | 2.27355 | NSCLC EMC ADC2264T | −2.76686 | NSCLC EMC TB0068tu | −1.64771 |
| SSC4 | −1.074 | NSCLC EMC 2407N | 1.01082 | NSCLC EMC SCC2279N | 1.81092 | NSCLC EMC ADC2270T | −1.71967 | NSCLC EMC 2388T | –1.999 |
| SSC5 | −0.917 | NSCLC EMC 2409N | 1.83913 | NSCLC EMC SCC2283N | 1.60123 | NSCLC EMC ADC2272T | −1.35225 | NSCLC EMC TB0003tu | −0.874497 |
| SSC6 | −0.85 | NSCLC EMC 2411N | 2.13296 | NSCLC EMC SCC2287N | 1.25768 | NSCLC EMC ADC2294T | −1.64306 | NSCLC EMC TB0007tu | −1.78151 |
| SSC7 | −0.586 | NSCLC EMC 2419N | 1.3914 | NSCLC EMC SCC2299N | 2.25152 | NSCLC EMC ADC2296T | 1.05778 | NSCLC EMC TB0008tu | −2.41861 |
| SSC8 | −0.222 | NSCLC EMC ADC2231N | 1.73391 | NSCLC EMC SCC2301N | 1.69104 | NSCLC EMC ADC2308T | −0.189866 | NSCLC EMC TB0024tu | −1.28513 |
| SSC9 | −0.218 | NSCLC EMC ADC2245N | 1.67082 | NSCLC EMC SCC2305N | −0.178562 | NSCLC EMC ADC2310T | −1.63799 | NSCLC EMC TB0030tu | −1.50773 |
| SSC10 | −0.031 | NSCLC EMC ADC2247N | 1.44294 | NSCLC EMC SCC2313N | 1.565 | NSCLC EMC ADC2316T | 0.257932 | NSCLC EMC TB0035tu | −0.297382 |
| SSC11 | 0.4115 | NSCLC EMC ADC2255N | 1.33287 | NSCLC EMC SCC2317N | 0.479814 | NSCLC EMC ADC2326T | 1.72582 | NSCLC EMC TB0058tu | 0.498912 |
| SSC12 | 2.183 | NSCLC EMC ADC2257N | 1.76375 | NSCLC EMC SCC2321N | 1.7578 | NSCLC EMC ADC2340T | −1.28645 | NSCLC EMC TB0063tu | −1.82753 |
|  |  | NSCLC EMC ADC2263N | 2.5841 | NSCLC EMC TB0009he | 2.6547 | NSCLC EMC ADC2347T | −0.220986 | NSCLC EMC 2406T | −1.2258 |
|  |  | NSCLC EMC ADC2269N | 1.52395 | NSCLC EMC TB0010he | −0.355598 | NSCLC EMC ADC2348T | −1.005 | NSCLC EMC 2412T | −0.573821 |
|  |  | NSCLC EMC ADC2277N | 1.81775 | NSCLC EMC TB0011he | 2.09784 | NSCLC EMC LCC2218T | −0.363102 | NSCLC EMC ADC2278T | −2.78778 |
|  |  | NSCLC EMC ADC2293N | 1.67249 | NSCLC EMC TB0017he | 1.29606 | NSCLC EMC LCC2234T | −2.93679 | NSCLC EMC LCC2228T | −2.04087 |
|  |  | NSCLC EMC ADC2295N | 1.23016 | NSCLC EMC TB0022he | 2.35733 | NSCLC EMC SCC2288T | 0.0375049 | NSCLC EMC LCC2282T | −1.7879 |
|  |  | NSCLC EMC ADC2307N | 2.14162 | NSCLC EMC TB0028tu | 0.252656 | NSCLC EMC TB0006tu | −2.61191 | NSCLC EMC SCC2208T | –3.00304 |
|  |  | NSCLC EMC ADC2315N | 2.43815 | NSCLC EMC TB0031he | 1.39732 | NSCLC EMC TB0009tu | −1.34717 | NSCLC EMC SCC2214T | −1.8942 |
|  |  | NSCLC EMC ADC2320N | 2.2265 | NSCLC EMC TB0033he | 1.46947 | NSCLC EMC TB0013tu | −0.727857 | NSCLC EMC SCC2222T | −0.868268 |
|  |  | NSCLC EMC ADC2325N | 1.03611 | NSCLC EMC TB0036he | 1.25234 | NSCLC EMC TB0015tu | −2.39792 | NSCLC EMC SCC2236T | −2.59354 |
|  |  | NSCLC EMC ADC2330N | 1.8243 | NSCLC EMC TB0050he | 1.63648 | NSCLC EMC TB0018tu | −1.24647 | NSCLC EMC SCC2252T | −2.70155 |
|  |  | NSCLC EMC LCC2217N | 2.17596 | NSCLC EMC TB0053he | 2.23907 | NSCLC EMC TB0019tu | −1.80538 | NSCLC EMC SCC2280T | −1.51872 |
|  |  | NSCLC EMC LCC2233N | 1.51733 | NSCLC EMC TB0067he | 0.912122 | NSCLC EMC TB0025tu | −1.3504 | NSCLC EMC SCC2298T | −2.21122 |
|  |  | NSCLC EMC LCC2249N | 1.60061 | NSCLC EMC TB0068he | 1.20162 | NSCLC EMC TB0027tu | 1.57824 | NSCLC EMC SCC2302T | −1.67035 |
|  |  | NSCLC EMC LCC2281N | 1.71351 | NSCLC EMC 2323N | 0.405839 | NSCLC EMC TB0031tu | −1.46625 | NSCLC EMC SCC2306T | −0.767736 |
|  |  | NSCLC EMC LCC2303N | 3.16775 | NSCLC EMC 2384T | −0.859729 | NSCLC EMC TB0033tu | −2.9327 | NSCLC EMC SCC2318T | −2.24341 |
|  |  | NSCLC EMC LCC2311N | 1.95097 | NSCLC EMC 2408T | −1.70654 | NSCLC EMC TB0034tu | 0.341961 | NSCLC EMC SCC2341T | −2.98242 |
|  |  | NSCLC EMC LCC2327N | 2.075 | NSCLC EMC 2410T | −1.22272 | NSCLC EMC TB0037tu | −2.4405 | NSCLC EMC SCC2358T | −1.75585 |
